# HMGB1 couples LEF1 to regulate B cell immunity

**DOI:** 10.1172/jci.insight.187002

**Published:** 2025-09-23

**Authors:** Qiuyue Chen, Ziyin Zhang, Nanshu Xiang, Li Luo, Xin Dai, Danqing Kang, Lu Yang, Yingzi Zhu, Jiang Chang, Yukai Jing, Na Li, Qianglin Chen, Panpan Jiang, Ju Liu, Yanmei Huang, Heather Miller, Xinyuan Zhou, Fang Zheng, Quan Gong, Chaohong Liu

**Affiliations:** 1 Clinical Molecular Immunology Center, Department of Immunology, School of Medicine, Yangtze University, Jingzhou, China.; 2 Department of Pathogen Biology, School of Basic Medicine, Tongji Medical College and State Key Laboratory for Diagnosis and Treatment of Severe Zoonotic Infectious Diseases, Huazhong University of Science and Technology, Wuhan, China.; 3 Department of Rheumatology and Immunology, Tongji Hospital, Tongji Medical College, Huazhong University of Science and Technology, Wuhan, China.; 4 Department of Intracellular Pathogens, National Institute of Allergy and Infectious Diseases, NIH, Bethesda, Maryland, USA.; 5 Institute of Immunology, Army Medical University, Chongqing, China.; 6 Department of Immunology, Tongji Medical College, Huazhong University of Science and Technology, Wuhan, China.

**Keywords:** Cell biology, Development, Immunology, Signal transduction

## Abstract

Secreted high mobility group box protein 1 (HMGB1) regulates the adaptive immune response and acts as a biosensor for cells undergoing necrosis, stress, and inflammatory stimulation. However, its role in B cells remains enigmatic. Here, we demonstrate that HMGB1 is critical for peripheral B cell homeostasis and humoral immunity. Conditional deletion of *Hmgb1* in B cells led to expanded marginal zone B cells, reduced B1a cells, and impaired antigen-specific antibody responses. Mechanistically, HMGB1 deficiency enhanced proximal and distal B cell receptor (BCR) signaling, probably via increased CD21 expression, which lowered the BCR activation threshold. This phenotype was linked to reduced lymphoid enhancer-binding factor 1 (LEF1) levels, a Wnt-responsive transcription factor, as HMGB1 directly bound the *Lef1* promoter to sustain its transcription, thereby repressing *Cd21*. Furthermore, HMGB1 constrained actin reorganization by suppressing the MST1/DOCK8/WASP axis, which feedback-modulated BCR clustering and signalosome recruitment. Collectively, HMGB1 ensures optimal BCR signaling by transcriptionally and cytoskeletally tuning activation thresholds, highlighting its dual role as a nuclear regulator and cytoskeletal modulator in B cell immunity.

## Abstract 2

The HMGB1-LEF1-CD21 axis regulates BCR signaling and MZ B cell differentiation.

## Graphical abstract

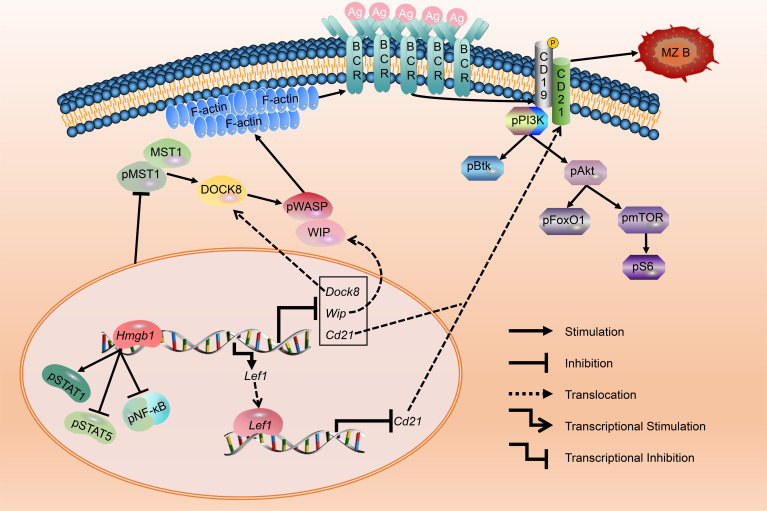

## Introduction

High mobility group box protein 1 (HMGB1) is a nuclear protein that plays a dual role as a DNA chaperone and a damage-associated molecular pattern (DAMP). It is secreted by necrotic and apoptotic cells and functions as a potent inducer of pro-inflammatory cytokine expression, thereby bridging innate and adaptive immune responses (1). Under conditions of severe cellular stress, HMGB1 translocates from the nucleus to the cytoplasm and is subsequently released into the extracellular milieu via lysosomal exocytosis or direct secretion. This extracellular HMGB1 triggers pro-inflammatory and immune-modulatory responses, highlighting its critical role in immune regulation (2).

While the role of HMGB1 in innate immune cells has been extensively studied, its function in B cells remains poorly understood. Recent studies have shed light on the interaction between HMGB1 and its receptor, the receptor for advanced glycation end products (RAGE). RAGE is expressed in peripheral B cells; however, its deficiency does not impair B cell development or activation following IgM and Toll-like receptor (TLR) stimulation (3). Intriguingly, activation of autoreactive B cells by chromatin and HMGB1/DNA immune complexes occurs independently of RAGE (3). Meanwhile, HMGB1 and RAGE have been shown to mediate TLR9-dependent activation through DNA-containing immune complexes, suggesting a nuanced role for HMGB1 in B cell activation (4). Furthermore, rituximab treatment in large B cell lymphoma inhibits STAT3 activity, leading to the release of nuclear HMGB1 and a subsequent reduction in IL-10 secretion, underscoring the therapeutic implications of HMGB1 modulation (5). Additionally, HMGB1 has been reported to activate B cells via TLR2 and CD36, further emphasizing its role in B cell signaling (6). Despite these advances, the consequences of intracellular HMGB1 deficiency in B cells remain unexplored, leaving a critical gap in our understanding of its immunoregulatory functions.

HMGB family proteins are known to interact with various transcription factors to modulate gene expression. For instance, HMGB2 binds to the LEF1 β-catenin complex, enhancing the expression of genes containing LEF1-binding sites (7). LEF1 binds to DNA via a single amino acid homology region, the HMG box, which is similar to nonhistone HMGB1 and several other transcriptional regulators (8). While B cell proliferation is enhanced following CD21 cross-linking by specific extracellular polyvalent ligands (9), CD21^lo^ human transitional B cells express elevated levels of LEF1 (10). Our prior research has demonstrated that LEF1 negatively regulates *Cd21* transcripts by binding to the *Cd21* promoter, thereby influencing B cell differentiation (11). However, it remains unclear whether HMGB1 modulates the transcription of *Cd21* through LEF1 to regulate B cell differentiation. Additionally, HMGB1 has been shown to activate the NF-κB signaling pathway via ERK in mouse embryonic fibroblasts, promoting cell migration (12). Whether HMGB1 interacts with NF-κB in B cells to regulate B cell activation remains an open question.

Actin dynamics are critical for B cell receptor (BCR) clustering and signalosome assembly. HMGB1 also plays a role in cytoskeletal dynamics by inducing actin reorganization in various cell types (13). In human umbilical vein endothelial cells, HMGB1 induces F-actin rearrangement through the phosphorylation of moesin at Thr558 (14). Conversely, in human lung cancer A549 cells, *HMGB1* deficiency enhances CREB phosphorylation, nuclear translocation, and activation by promoting the nuclear translocation of the PKA catalytic subunit, ultimately increasing β-actin polymerization via upregulation of neuronal Wiskott-Aldrich syndrome protein (N-WASP), a key regulator of actin cytoskeleton dynamics (15). Despite these findings, it is unknown whether HMGB1 regulates actin reorganization in B cells through similar mechanisms, which could provide critical feedback to BCR signaling.

Here, we identify HMGB1 as a critical regulator of peripheral B cell compartmentalization and activation thresholds. Using conditional *Hmgb1*-knockout (cKO) mice, we demonstrate that HMGB1 deficiency disrupts B cell homeostasis, leading to the aberrant expansion of marginal zone (MZ) B cells, a reduction in B1a cell populations, and impaired antigen-specific antibody responses. Mechanistically, HMGB1 constrains BCR signaling by both transcriptional and cytoskeletal pathways. At the transcriptional level, HMGB1 directly binds to the *Lef1* promoter to sustain LEF1 expression, which in turn represses *Cd21* transcription. Loss of HMGB1 results in reduced LEF1 level, leading to the upregulation of CD21 at both the transcription and protein levels. This disruption of the LEF1/CD21 axis likely contributes to the amplification of proximal and distal BCR signaling pathways, lowering the activation threshold and enhancing B cell responsiveness. Concurrently, HMGB1 modulates actin cytoskeletal dynamics by suppressing the mammalian sterile 20-like kinase 1 (MST1)/dedicator of cytokinesis 8 (DOCK8)/WASP axis, which regulates BCR clustering and signalosome assembly. In its absence, dysregulated actin reorganization enhances membrane spreading and sustained signaling microdomain formation. Together, these findings reveal HMGB1 as a multifaceted regulator of B cell function, integrating transcriptional control of activation thresholds with cytoskeletal modulation of receptor dynamics.

## Results

### HMGB1 is essential for differentiation of peripheral B cells and B1 cells, not for development of bone marrow B cells.

**Figure 1:**
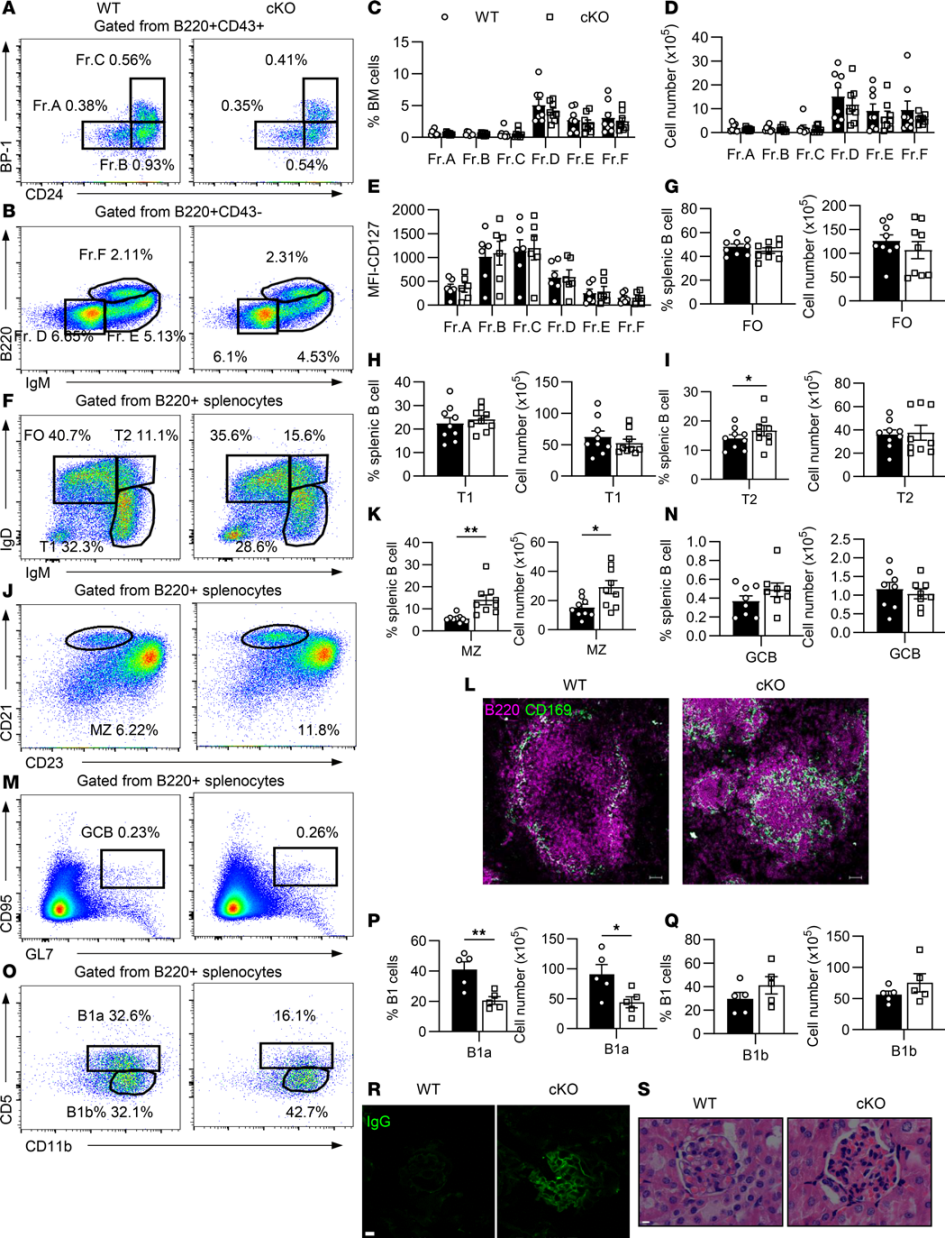
HMGB1 is essential for differentiation of peripheral B cells and B1 cells. (**A** and **B**) Flow cytometry analysis of bone marrow (BM) B cell subpopulations (pre-pro [Fr.A], pro [Fr.B], early-pre [Fr.C], late-pre [Fr.D], immature [Fr.E], and recirculating mature [Fr.F]) in WT and cKO mice. (**C** and **D**) Flow cytometry analysis for proportions and absolute numbers of total BM B cell subpopulations (*n* = 8). (**E**) Flow cytometry analysis of CD127 mean fluorescence intensity (MFI) in BM B cell subsets (*n* = 6). (**F**, **J**, and **M**) Flow cytometry analysis of splenic B cell subpopulations in WT and cKO mice. (**G**–**I**, **K**, and **N**) Flow cytometry analysis for proportions and absolute numbers of splenic B cell subsets (T1, T2, FO, MZ [*n* = 9]; GC [*n* = 8]). (**L**) Immunofluorescence staining for splenic MZ architecture (10× objective; scale bar = 50 μm). (**O**) Flow cytometry analysis of peritoneal B1a and B1b subsets. (**P**–**Q**) Flow cytometry analysis for proportions and numbers of peritoneal B1a/B1b cells (*n* = 5). (**R**) Immunofluorescence staining for renal glomeruli (60× objective; scale bar = 25 μm). (**S**) H&E staining for kidney sections (100× objective; scale bar = 20 μm). Shown are representative images of immunofluorescence and H&E staining from 1 of 3 independent experiments. Data are representative of 3 experiments. Multiple *t* tests (**C**–**E**) and 2-tailed unpaired Student’s *t* tests (**G**–**I**, **K**, **N**, **P**, and **Q**) were used for statistical analysis. Data shown as mean ± SEM. *P < 0.05, **P < 0.01.

The mRNA and protein levels of *Hmgb1* were obviously decreased in *Hmgb1*-cKO mice, *CD19Cre*^+/–^ *Hmgb1*^fl/fl^, confirming successful deletion of *Hmgb1* (Supplemental Figure 1, A and B; supplemental material available online with this article; https://doi.org/10.1172/jci.insight.187002DS1). To determine whether *Hmgb1* deficiency affects the development of bone marrow (BM) B cells, 6 BM B cell subsets (pre-pro, pro, early-pre, late-pre, immature, recirculating mature B cells) were analyzed by flow cytometry (gating, Supplemental Figure 6). No significant differences were observed in the proportions or numbers of these subsets between cKO and wild-type (WT; *CD19Cre*^+/–^ *Hmgb1*^fl/+^) mice, as well as the expression of CD127 on the surface of BM B cells (Figure 1, A–E). Furthermore, the total proportion and number of B220^+^ BM B cells remained unchanged in cKO mice (Supplemental Figure 1C). In contrast with prior studies revealing the role of HMGB1 in V(D)J recombination (16–19), we found no discernible differences in VH or Vκ chain recombination efficiency between WT and cKO mice (Supplemental Figure 1D). To further investigate potential defects in BCR assembly, we analyzed surface expression of Igμ (heavy chain) and Igκ/Igλ (light chains) across B cell subsets via flow cytometry. Consistent with the normal BM B cell development observed in cKO mice (Figure 1, C and D), there was no difference in Ig chain expression (Supplemental Figure 1, E–G). These findings suggest that *Hmgb1* deficiency has minimal impact on V(D)J recombination and BM B cell development in vivo.

In the spleen, the proportion and number of total B220^+^ B cells were comparable between WT and cKO mice (Supplemental Figure 1H). However, *Hmgb1* deficiency resulted in an increased proportion of T2 B cells, while the absolute number of T2 cells remained unchanged compared to controls. No significant differences were observed in the frequency or numbers of follicular (FO) or transitional type 1 (T1) B cells (Figure 1, F–I). In contrast, both the proportion and number of MZ B cells were markedly elevated in cKO mice (Figure 1, J and K). Immunofluorescence analysis further corroborated this expansion, revealing a pronounced enlargement of MZ B cell populations in cKO mice (Figure 1L). Despite these changes, germinal center (GC) B cell levels remained normal in cKO mice (Figure 1, M and N). Given that HMGB1 enhances the chemotactic activity of CXCL12 (20), a chemokine critical for B cell migration and localization, we assessed whether HMGB1 deficiency alters B cell responsiveness to CXCL12. Splenic B cells from cKO mice showed no significant impairment in migration efficiency compared to WT cells (Supplemental Figure 1I), suggesting that the altered MZ distribution is independent of CXCL12-driven migratory capacity.

Notably, the proportion and number of peritoneal B1a cells were reduced in cKO mice, whereas B1b cells remained unchanged (Figure 1, O–Q). Similarly, no differences were observed in the proportion or number of CD19^+^ B1 cells between WT and cKO mice (Supplemental Figure 1J). Interestingly, glomerular IgG deposits and immune cell infiltration were evident in the kidneys of cKO mice, suggesting a pronounced autoimmune response (Figure 1, R and S). In contrast, no abnormalities were observed in the colon, lungs, or liver of these mice (Supplemental Figure 1K).

Together, these findings demonstrate that HMGB1 plays a critical role in the differentiation of MZ B and B1a cells but does not appear to influence the development of early B cells in the BM.

### HMGB1 is involved in BCR activation and downregulates the proximal BCR signaling.

**Figure 2:**
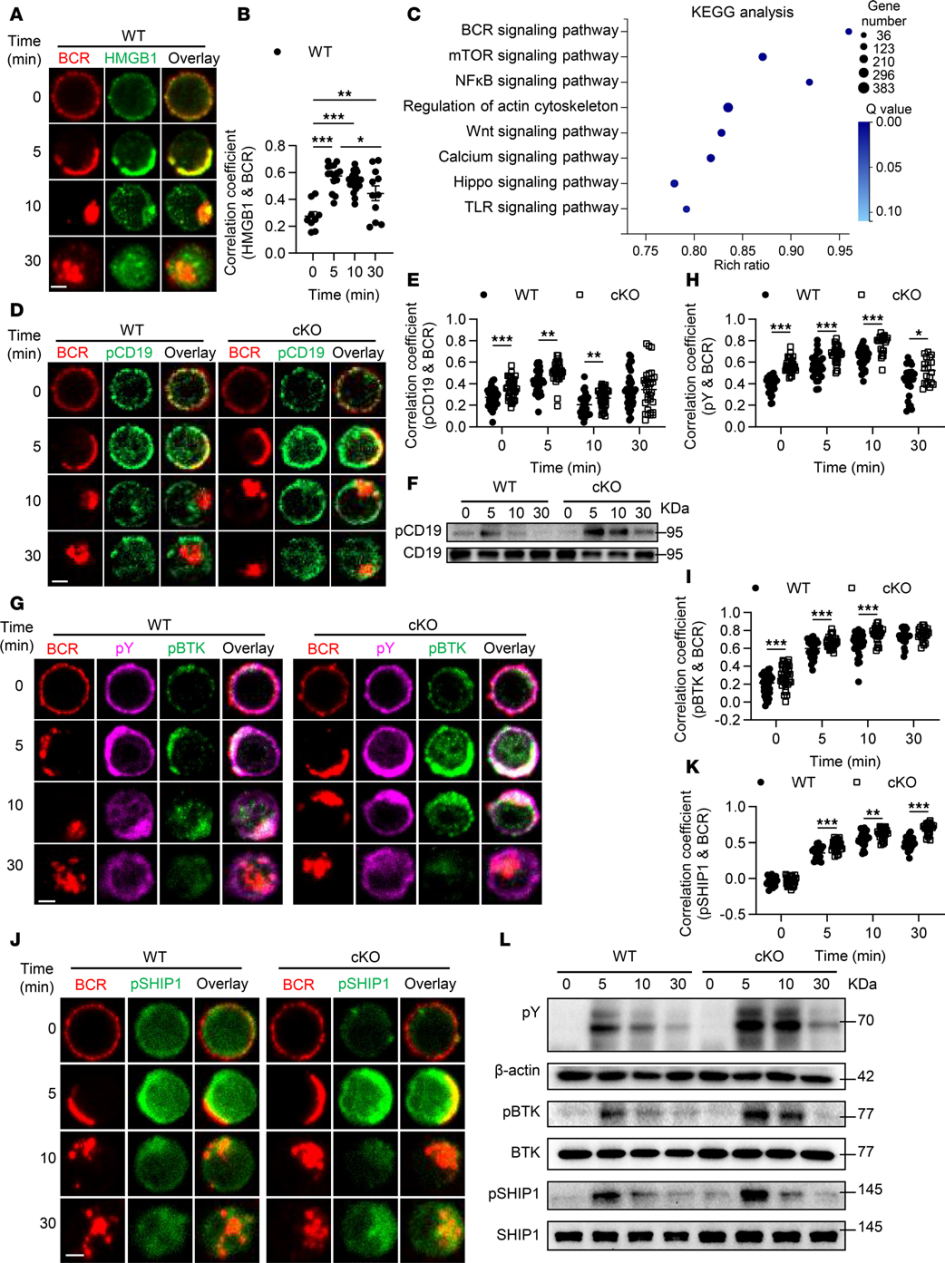
HMGB1 modulates BCR activation and proximal signaling. (**A**, **D**, **G**, and **J**) Confocal microscopy of HMGB1, phosphorylated CD19 (p-CD19), phosphorylated tyrosine (p-Y), p-BTK, and p-SHIP1 in splenic B cells stimulated with anti-BCR (60× objective; scale bar = 2.5 μm). (**B**, **E**, **H**, **I**, and **K**) Confocal microscopy for Pearson’s correlation coefficients between BCR and signaling molecules (analyzed with NIS-Elements AR 3.2). (**C**) KEGG pathway enrichment of differentially expressed genes in cKO B cells (bubble size: gene count; color: *Q* value). (**F** and **L**) Western blotting of p-CD19, p-Y, p-BTK, and p-SHIP1 in anti-BCR–stimulated B cells (β-actin/total protein served as loading controls). Representative confocal microscopy image for 3 independent experiments. Data are representative of 3 experiments. One-way ANOVA (**B**) and multiple *t* tests (**E**, **H**, **I**, and **K**) were used for statistical analysis. Data shown as mean ± SEM. *P < 0.05, **P < 0.01, ***P < 0.001.

B cell differentiation is primarily governed by B cell activation and BCR signaling. To assess whether HMGB1 is involved in BCR activation, we examined the spatiotemporal relationship between BCR and HMGB1 using confocal microscopy. Notably, this colocalization was markedly elevated at 5, 10, and 30 minutes poststimulation compared with the resting state (Figure 2, A and B), indicating that HMGB1 is highly spatially associated with the BCR signalosome and might regulate the BCR signaling activation. To preliminarily explore this, we performed bulk RNA-sequencing analysis on B cells sorted from WT and cKO mice. By analyzing the Kyoto Encyclopedia of Genes and Genomes (KEGG) pathways with differentially expressed genes identified from these cells, we observed enrichment of multiple signaling pathways, such as BCR, mTOR, NF-κB, Wnt, calcium, Hippo, and TLR signaling (Figure 2C). Among these pathways, HMGB1 has been previously implicated in mTOR, NF-κB, Wnt, calcium, Hippo, and TLR signaling pathways (12, 21–24), revealing that our transcriptional data are highly consistent with existing literature. Therefore, we hypothesized that HMGB1 may also play a role in BCR signaling. To address this, we first evaluated the phosphorylation status of CD19, an upstream regulator of BCR signaling. Surprisingly, both the expression of p-CD19 and colocalization between p-CD19 and BCR were significantly increased in cKO B cells after stimulation (Figure 2, D–F). To assess the overall BCR signaling activity, we examined the spatiotemporal relationship of p-Y proteins and the phosphorylation of BTK, a direct downstream effector of CD19. Both p-Y and p-BTK showed enhanced colocalization with BCR in cKO B cells (Figure 2, G–I). Since BTK activation is negatively regulated by SHIP1, a proximal inhibitor of BCR signaling, we then explored the impact of HMGB1 deficiency on SHIP1 activation. Colocalization between p-SHIP1 and BCR was significantly increased at 5, 10, and 30 minutes in cKO B cells (Figure 2, J and K). Immunoblotting further verified that the expression levels of p-Y, p-BTK, and p-SHIP1 were all elevated in cKO B cells (Figure 2L). Collectively, these results indicate that HMGB1 acts as a negative regulator of proximal BCR signaling.

**Figure 3:**
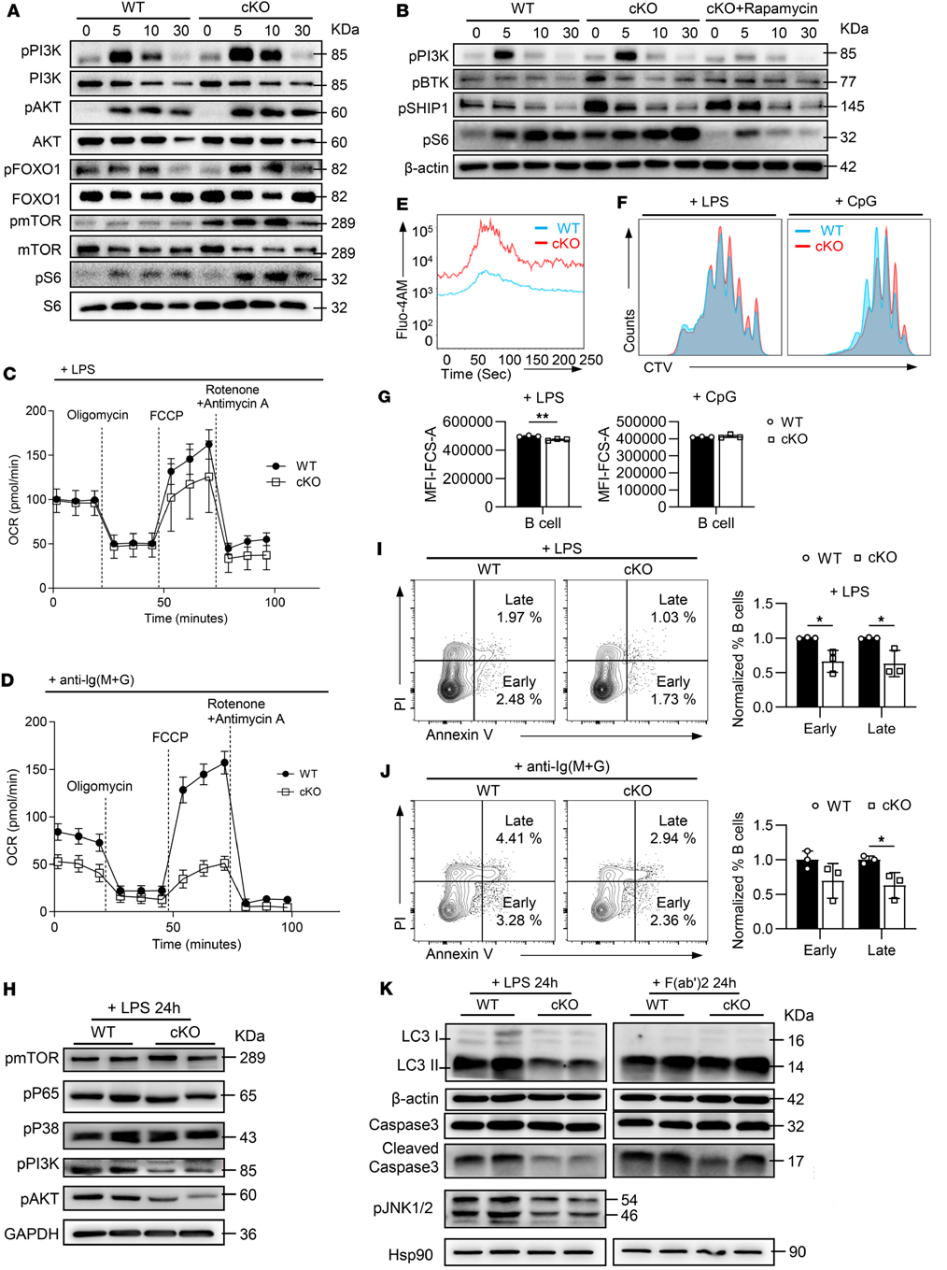
*Hmgb1* deficiency enhances anti-BCR–stimulated PI3K/AKT/mTOR signaling while impairing the LPS-induced TLR signaling and cell apoptosis. (**A**) Western blotting of p-PI3K, p-AKT, p-FOXO1, p-mTOR, and p-S6 in anti-BCR–stimulated B cells. (**B**) Western blotting of p-PI3K, p-BTK, p-SHIP1, and p-S6 in anti-BCR–stimulated B cells following rapamycin pretreatment in vitro. (**C** and **D**) OCR of LPS- or anti-BCR–stimulated splenic B cells. (**E**) Flow cytometry analysis for calcium flux in anti-BCR-stimulated B cells. (**F** and **G**) Flow cytometry analysis for proliferation (CellTrace Violet [CTV] dilution) and FSC-A MFI of LPS- or CpG-stimulated B cells. (**H**) Western blotting of p-mTOR, p–NF-κB, p-P38, p-PI3K, and p-AKT in B cells stimulated by LPS for 24 hours. (**I**–**K**) Apoptosis (flow cytometry) and LC3 I/II, caspase-3, or p-JNK1/2 expression (Western blotting) in LPS- or anti-BCR–stimulated B cells. PI, propidium iodide. Data are representative of 3 experiments. Multiple *t* tests (**C**, **D**, **I**, and **J**) and 2-tailed unpaired Student’s *t* test (**G**) were used for statistical analysis. Data shown as mean ± SEM. *P < 0.05, **P < 0.01.

HMGB1 has been shown to play a role in the PI3K/AKT signaling pathway in other cell types. Additionally, transcriptional data also suggested alterations in mTOR signaling in cKO B cells (Figure 2C). We next explored the impact of HMGB1 deficiency on the activation of these pathways in BCR signaling following anti-BCR stimulation. Immunoblotting revealed increased expressions of p-PI3K, p-AKT, p-FOXO1, p-mTOR, and p-S6 in cKO B cells (Figure 3A). These findings indicate that the HMGB1 deficiency in B cells results in the overactivated PI3K/AKT/mTOR signaling upon anti-BCR stimulation. Notably, treatment with rapamycin, an mTOR inhibitor, restored BCR signaling in cKO B cells, as evidenced by the recovery of p-PI3K, p-BTK, and p-SHIP1 levels (Figure 3B). This reveals mTOR exerts positive feedback on BCR signaling in cKO B cells.

Given the close relationship between mTOR activity and cellular metabolism, we assessed the impact of *Hmgb1* deficiency on B cell oxidative phosphorylation using real-time Seahorse assays. The maximum oxygen consumption rate (OCR) was markedly lower in cKO B cells compared with WT cells (Figure 3, C and D). As a functional readout of BCR signaling, we measured calcium flux in B cells following antigenic stimulation and observed an increase in cKO B cells (Figure 3E). We also compared the proliferative capacity of WT and cKO B cells in response to LPS or CpG stimulation. While proliferation was comparable between the 2 groups under both stimuli, cKO B cells were significantly smaller than WT cells following LPS stimulation (Figure 3, F and G), suggesting aberrant signal transduction in cKO B cells. To further investigate this, we analyzed the phosphorylation of key molecules involved in cell cycle progression, protein synthesis, and cellular growth after 24 hours of LPS stimulation. Western blot analysis revealed no obvious differences in p-mTOR, p-P65, or p-P38 (Figure 3H). Interestingly, phosphorylation of JNK1/2, PI3K, and AKT was substantially reduced in LPS-stimulated cKO B cells (Figure 3, H and K). These data suggest that *Hmgb1*-deficient B cells exhibit impaired responses to LPS stimulation, including reduced phosphorylation of PI3K, AKT, and JNK1/2, which may lead to diminished cellular growth. However, the weakened activation of these molecules did not impair proliferative capacity, implying compensatory mechanisms. To clarify this point, we assessed the apoptosis in WT and cKO B cells with LPS and anti-BCR stimulation, given prior reports linking HMGB1 to autophagy and apoptosis (25). Strikingly, the apoptosis of cKO B cells was significantly decreased (Figure 3, I and J). Corroborating this, we observed decreased expressions of LC3 (a marker of autophagic flux) and cleaved caspase-3 (an apoptosis executor) in cKO B cells (Figure 3K). Together, these results indicate that while LPS-stimulated cKO B cells exhibit reduced activation of p-PI3K, p-AKT, and p-JNK1/2, their proliferative capacity remains intact, likely due to a concurrent decrease in apoptosis.

### Hmgb1 deficiency positively regulates the activation of NF-κB signaling.

**Figure 4:**
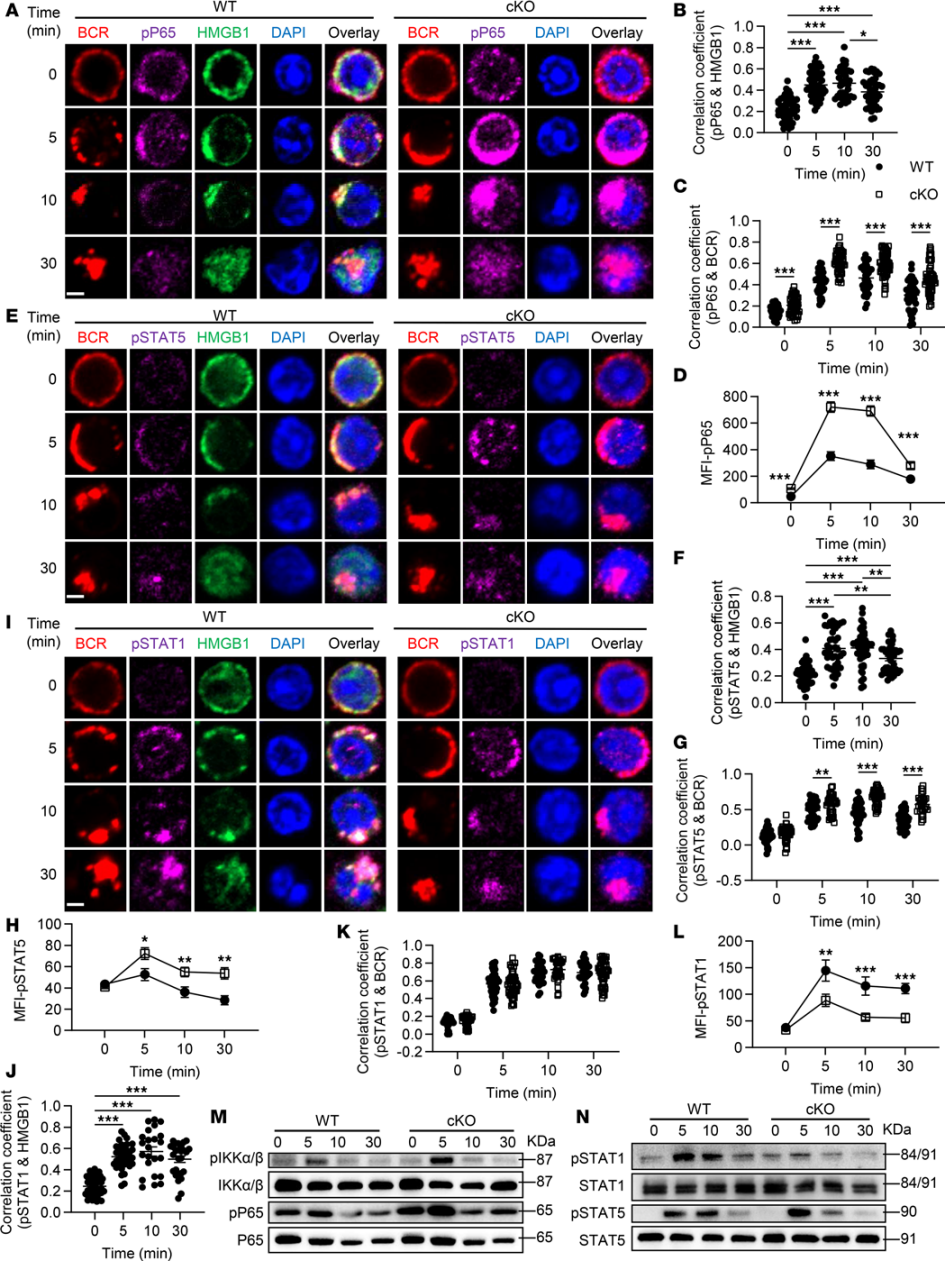
*Hmgb1* deficiency enhances the activation of NF-κB and STAT5 while impairing the activation of STAT1. (**A**, **E**, and **I**) Confocal microscopy of p-P65, p-STAT5, p-STAT1, and HMGB1 in splenic B cells stimulated with anti-BCR (60× objective; scale bar = 2.5 μm). (**B**, **C**, **F**, **G**, **J**, and **K**) Confocal microscopy for Pearson’s correlation coefficients between BCR, HMGB1, and signaling molecules. (**D**, **H**, and **L**) Confocal microscopy for MFI of p-P65, p-STAT5, and p-STAT1. (**M** and **N**) Western blotting of p-IKKα/β, p-P65, p-STAT1, and p-STAT5 in splenic B cells stimulated with anti-BCR. Representative confocal microscopy image for 3 independent experiments. One-way ANOVA (**B**, **F**, and **J**) and multiple *t* tests (**C**, **D**, **G**, **H**, **K**, and **L**) were used for statistical analysis. Data shown as mean ± SEM. *P < 0.05, **P < 0.01, ***P < 0.001.

NF-κB, a signaling intermediate located distally in both BCR and TLR4 pathways, has been demonstrated to be hyperactive in other models of autoimmunity (26). Additionally, HMGB1 has been established as a key regulator of the NF-κB signaling pathway in non-B cells, a finding corroborated by our KEGG analysis. To validate this phenotype of *Hmgb1*-deficient B cells, we investigated the spatiotemporal relationship between p-P65 and HMGB1 or the BCR. In WT B cells stimulated with anti-BCR, the correlation coefficients between HMGB1 and p-P65, p-STAT5, or p-STAT1 peaked at 10 minutes and exhibited a slight decline by 30 minutes (Figure 4, A, B, E, F, I, and J). These patterns align with the dynamic natures of NF-κB and STAT signaling, which often exhibit transient activation followed by feedback-driven downregulation. This observation suggests that HMGB1 can function as a signaling transducer within BCR signalosomes to modulate downstream events. Notably, in activated cKO B cells, colocalization of p-P65 or p-STAT5 with the BCR was significantly enhanced, accompanied by markedly elevated MFI of p-P65 and p-STAT5 (Figure 4, C, D, G, and H). Consistent with this, immunoblotting demonstrated increased levels of p-IKKα/β, p-P65, and p-STAT5 in cKO B cells (Figure 4, M and N), collectively indicating that HMGB1 suppresses NF-κB and STAT5 signaling. In contrast, the colocalization of p-STAT1 with the BCR remained unaltered in cKO B cells (Figure 4K), while the MFI of p-STAT1 decreased after BCR stimulation (Figure 4L). Immunoblotting further verified reduced p-STAT1 levels in cKO B cells (Figure 4N), a trend opposing that observed for p-P65 and p-STAT5. Taken together, our findings suggest a unique regulatory mechanism whereby HMGB1 modulates STAT1 activation, distinct from its suppressive role in regulating other transcription factors downstream of BCR signaling, such as NF-κB and STAT5.

### Hmgb1 deficiency enhances the accumulation of F-actin via the MST1/DOCK8/WASP axis.

**Figure 5:**
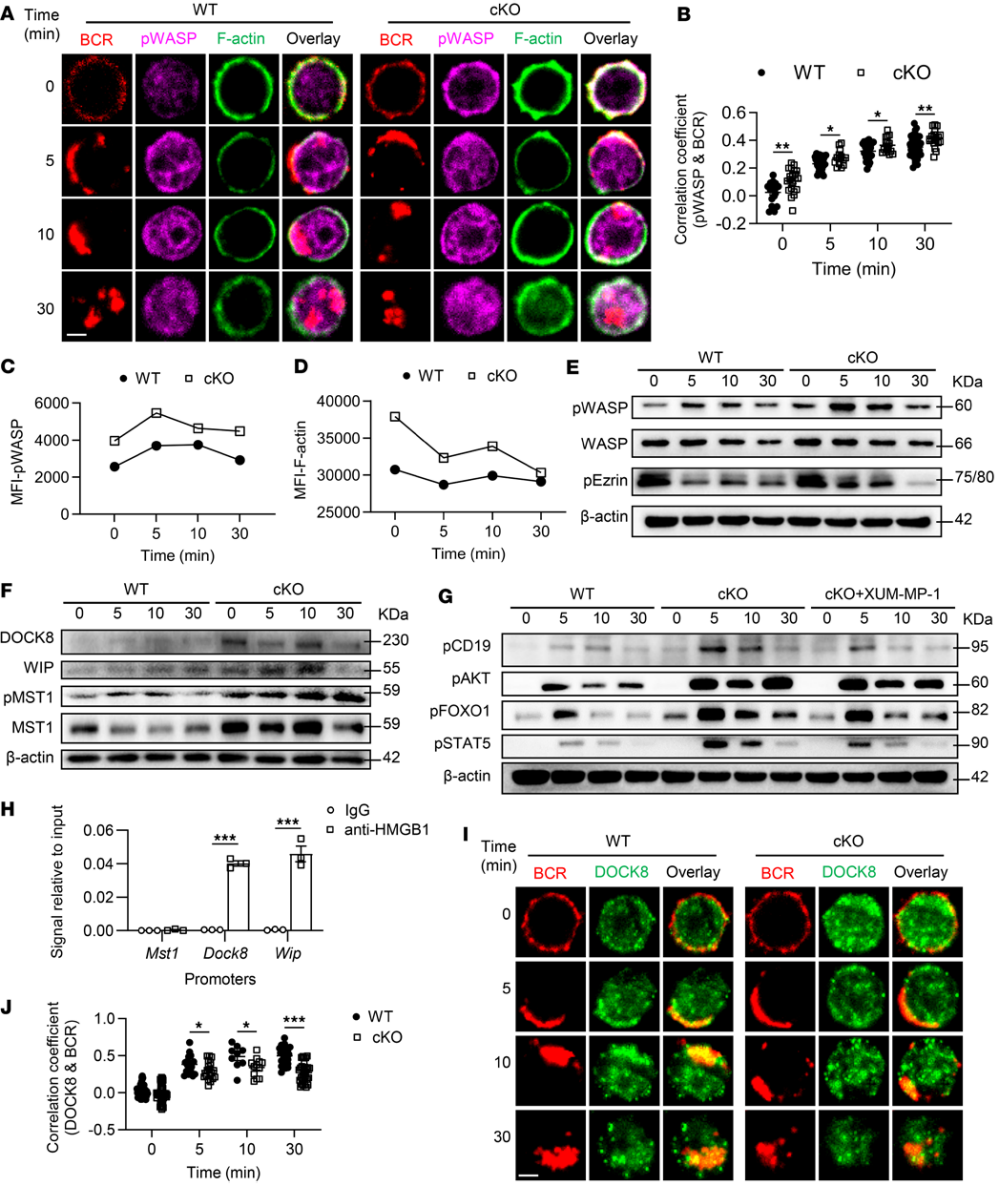
*Hmgb1* deficiency promotes F-actin accumulation via the MST1/DOCK8/WASP axis. (**A** and **I**) Confocal microscopy of p-WASP, F-actin, and DOCK8 in splenic B cells stimulated with anti-BCR (60× objective; scale bar = 2.5 μm). Representative confocal microscopy image for 3 independent experiments. (**B** and **J**) Pearson’s coefficients between BCR and p-WASP or DOCK8 analyzed by confocal microscopy. (**C** and **D**) Phos flow analysis of p-WASP and F-actin MFI in anti-BCR–stimulated B cells. (**E** and **F**) Western blotting of p-WASP, WASP, p-Ezrin, WIP, DOCK8, p-MST1, and MST1 in anti-BCR–stimulated B cells. (**G**) Western blotting of p-CD19, p-AKT, p-FOXO1, DOCK8, and p-STAT5 in anti-BCR–stimulated B cells following XMU-MP-1 pretreatment in vitro. (**H**) ChIP-RT-PCR of HMGB1 binding to *Mst1*, *Wip*, and *Dock8* promoters in splenic B cells. Data are representative of 3 experiments. Multiple *t* tests (**B**, **H**, and **J**) were used for statistical analysis. Data shown as mean ± SEM. *P < 0.05, **P < 0.01, ***P < 0.001.

Besides the signaling pathways we addressed above, we also observed that cKO B cells developed an abnormal regulation of actin cytoskeleton based on our RNA-Seq data (Figure 2C). Actin dynamics plays a crucial role in BCR signaling, as actin reorganization is essential for the formation of the BCR signalosome, which is critical for B cell activation and downstream responses. Upon BCR engagement, actin polymerization and depolymerization are tightly regulated to facilitate BCR clustering, signal transduction, and cell spreading (27, 28). To demonstrate whether HMGB1 could modulate actin reorganization to facilitate BCR signaling, we examined the spatiotemporal relationship between BCR and p-WASP, a key actin nucleation factor, in WT and cKO B cells upon anti-BCR treatment. *Hmgb1* deficiency enhanced the colocalization of p-WASP with BCR, and both the expression of p-WASP and F-actin showed higher MFI in cKO B cells, as measured by phos flow (Figure 5, A–D). Consistently, immunoblotting revealed the same increased level of p-WASP proteins in cKO B cells following anti-BCR stimulation (Figure 5E). p-Ezrin was also altered, with elevated levels at 0, 5, and 10 minutes but reduced levels at 30 minutes in cKO B cells (Figure 5E).

To further explore the upstream regulators of WASP activation, we examined the expression of DOCK8 and WASP-interacting protein (WIP; a scaffolding protein that stabilizes WASP and promotes its activation in actin cytoskeleton signaling), as well as MST1, which influences DOCK8 activity, in WT and cKO B cells following anti-BCR stimulation. The levels of DOCK8, WIP, MST1, and p-MST1 were all elevated in cKO B cells (Figure 5F). To identify which actin regulator mediates feedback to BCR signaling, we used various inhibitors and found that specifically inhibiting MST1 with XMU-MP-1 rescued BCR signaling, as evidenced by restored levels of p-CD19, p-AKT, p-FOXO1, and p-STAT5 (Figure 5G). Given that HMGB1 is involved in chromatin remodeling and the regulation of gene transcription, we next assessed whether HMGB1 could regulate the transcription of *Mst1*, *Dock8*, and *Wip* by ChIP followed by real-time PCR (ChIP-RT-PCR) assay. HMGB1 strongly bound to the promoters of *Wip* and *Dock8* but not *Mst1* (Figure 5H). Furthermore, the spatiotemporal relationship between BCR and DOCK8 was disrupted in cKO B cells, as indicated by a significantly reduced correlation coefficient (Figure 5, I and J). These results suggest that HMGB1 negatively regulates actin reorganization through the MST1/DOCK8/WASP axis, with MST1 providing feedback to BCR signaling.

### Hmgb1 deficiency enhances B cell spreading, BCR clustering, and BCR signalosome recruitment in response to membrane antigens.

**Figure 6:**
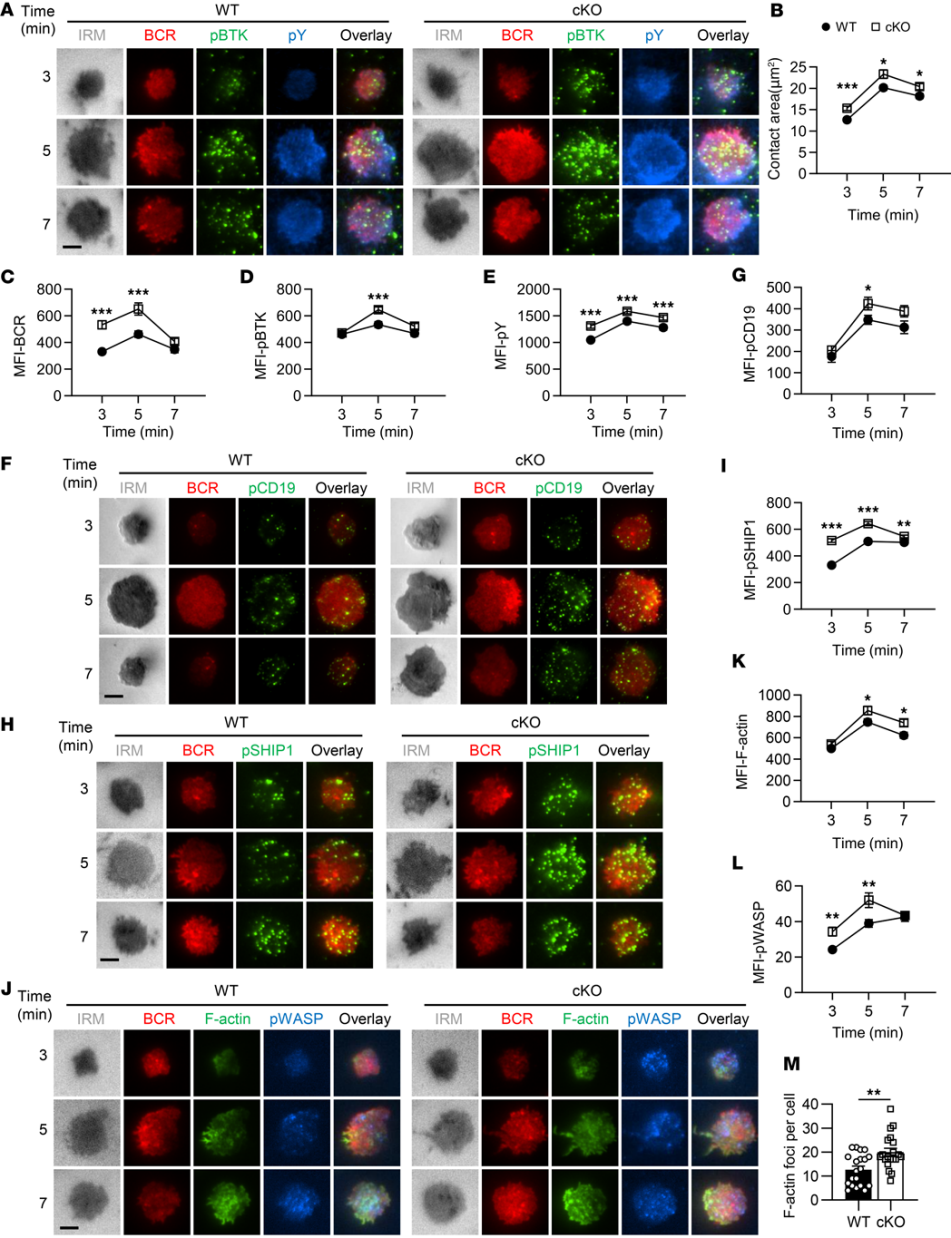
*Hmgb1* deficiency enhances B cell spreading and BCR signalosome recruitment. (**A**, **F**, **H**, and **J**) Total internal reflection fluorescence microscopy (TIRFm) of p-BTK, p-Y, p-CD19, p-SHIP1, F-actin, and p-WASP in splenic B cells stimulated with anti-BCR (60× objective; scale bar = 2.5 μm). (**B**) Quantification of B cell contact area analyzed by TIRFm. (**C**–**E**, **G**, **I**, **K**, and **L**) TIRFm for MFI of BCR, p-BTK, p-Y, p-CD19, p-SHIP1, F-actin, and p-WASP in anti-BCR–stimulated B cells from 3 independent experiments. (**M**) F-actin foci in anti-BCR–stimulated B cells quantified by ImageJ (NIH). Data analyzed with NIS-Elements AR 3.2. Representative of 3 experiments. Multiple *t* tests (**B**–**E**, **G**, **I**, **K**, and **L**) and 2-tailed unpaired Student’s *t* tests (**M**) were used for statistical analysis. Data shown as mean ± SEM. *P < 0.05, **P < 0.01, ***P < 0.001.

To investigate the impact of *Hmgb1* deletion on early B cell activation events, we utilized liposome-based artificial membranes conjugated with membrane antigens (mAg) to mimic the dynamic phospholipid bilayer of cell membranes in vitro. The spreading of B cells was measured by the contact area, while BCR clustering was calculated by MFI of BCR in the contact area. Compared with WT, cKO B cells exhibited significantly increased contact area and enhanced BCR aggregation (Figure 6, A–C). Furthermore, accumulation of signalosome components, including p-Y, p-BTK, p-CD19, and p-SHIP1, within the contact area was markedly elevated in cKO B cells (Figure 6, D–I). Recruitment of p-WASP and F-actin to the contact area was also substantially enhanced in cKO B cells, with approximately 20 F-actin foci per cell compared with WT (Figure 6, J–M). This heightened F-actin formation correlated with increased cell spreading and pronounced F-actin polymerization at the leading edge of cKO B cells. Together, these results demonstrate that *Hmgb1* deficiency amplifies early B cell activation and actin recruitment in response to mAg stimulation, aligning with the broader hyperactivation of BCR signaling observed in cKO B cells.

### HMGB1 is indispensable for T-independent and T-dependent humoral immune responses.

**Figure 7:**
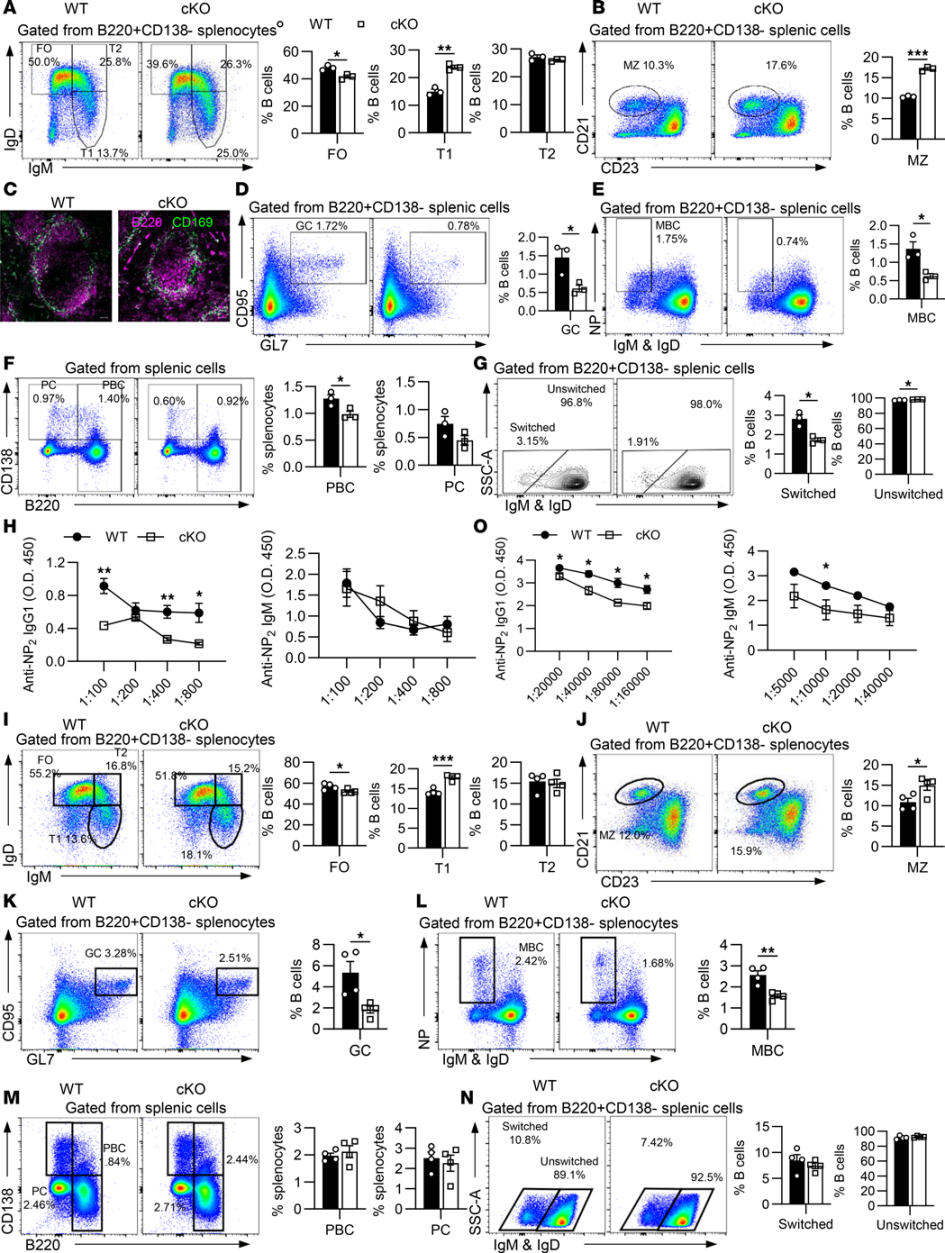
HMGB1 participates in both the T-independent and T-dependent immune response. (**A**, **B**, and **D**–**G**) Flow cytometry of splenic subsets in NP-Ficoll–immunized mice (T-independent [TI] response; *n* = 3). (**I**–**N**) Flow cytometry of splenic subsets after secondary NP-KLH immunization (T-dependent [TD] response; *n* = 4). (**C**) Immunofluorescence staining of splenic MZ (10× objective; scale bar = 50 μm). Shown are representative images of immunofluorescence from 1 of 3 independent experiments. (**H** and **O**) ELISA of NP-IgM and NP-IgG1 titers in TI- or TD-immunized mice. Two-tailed unpaired Student’s *t* tests (**A**, **B**, **D**–**G**, and **I**–**N**) and multiple *t* tests (**H** and **O**) were used for statistical analysis. Data shown as mean ± SEM. *P < 0.05, **P < 0.01, ***P < 0.001.

Innate-like B cells, such as MZ and B1 B cells, serve as frontline defenders in humoral immunity, enabling rapid containment of T-independent (TI) pathogens and priming adaptive immune pathways for sustained protection. Having seen the significant changes of MZ and B1a B cell populations in cKO mice (Figure 1, K and P), we hypothesized impaired TI humoral immunity in these mice. To test this, WT and cKO mice were immunized with the TI antigen NP-Ficoll. Postimmunization, cKO mice exhibited significant expansion of T1 and MZ B cells alongside a reduction in FO B cells (Figure 7, A and B). Immunofluorescence analysis further revealed MZ enlargement in cKO spleens (Figure 7C). Strikingly, GC B cells, plasmablast cells (PBCs), memory B cells (MBCs), and antibody class-switching efficiency were all diminished in cKO mice, with plasma cells (PCs) showing a downward trend (Figure 7, D–G). Despite comparable total cell numbers across subsets (Supplemental Figure 2, A–F), cKO sera displayed reduced NP-specific IgG1 but normal IgM levels (Figure 7H), aligning with defective class-switching. Together, these findings demonstrate HMGB1 is critical for TI humoral responses.

To assess HMGB1’s role in TD immunity, mice were immunized with NP-KLH. After primary immunization, cKO mice showed elevated MZ B cells (Supplemental Figure 3, A–H), reduced PBCs (Supplemental Figure 3, I and J), paradoxically increased PCs (Supplemental Figure 3K), and unchanged MBCs (Supplemental Figure 3, L and M). While NP-specific IgM was reduced, IgG1 remained unchanged (Supplemental Figure 3, N and O), suggesting compromised IgM-producing PC function. Upon secondary TD immunization, cKO mice mirrored TI response defects with declined FO, GC, and MBC frequencies yet expanded T1 and MZ B cells (Figure 7, I–L). Although PBCs, PCs, and class-switching metrics were unaffected, NP-specific IgM and IgG1 production were both impaired following the secondary immunization (Figure 7, M–O). There was only an obvious decrease of cell numbers in FO and GC B cells in cKO mice (Supplemental Figure 4, A–F). These deficits of humoral immune responses in cKO mice were independent of T cell help, evidenced by the comparable proportion of T follicular helper cells in WT and cKO mice (Supplemental Figure 4G).

Previous studies have implicated HMGB1 in regulating the expressions or functions of activation-induced cytidine deaminase (AID), CD40, and MHC-II (21, 29, 30), key players for the B cell differentiation and function in immune response. Therefore, we also detected expressions of these proteins in both groups of mice. While CD40 expression was normal in cKO B cells (Supplemental Figure 4H), MHC-II and AID levels were modestly elevated after secondary TD immunization (Supplemental Figure 4, I and J). Anti-BCR stimulation further revealed HMGB1 suppresses AID via BCR-dependent pathways (Supplemental Figure 4K). Given HMGB1’s known role in dampening NF-κB and MAPK signaling in B cells following anti-BCR stimulation (Figure 4, C and M, and Supplemental Figure 4K), critical for AID and MHC-II induction, we propose HMGB1 fine-tunes these pathways to prevent excessive activation. However, these molecular changes contribute minimal effects to impaired TD-immune responses in cKO mice.

Collectively, these results indicate that HMGB1 is necessary for effective humoral immune responses induced by both TD and TI antigens.

### HMGB1 interacts with LEF1 to negatively regulate Cd21 expression, potentially suppressing B cell activation.

**Figure 8:**
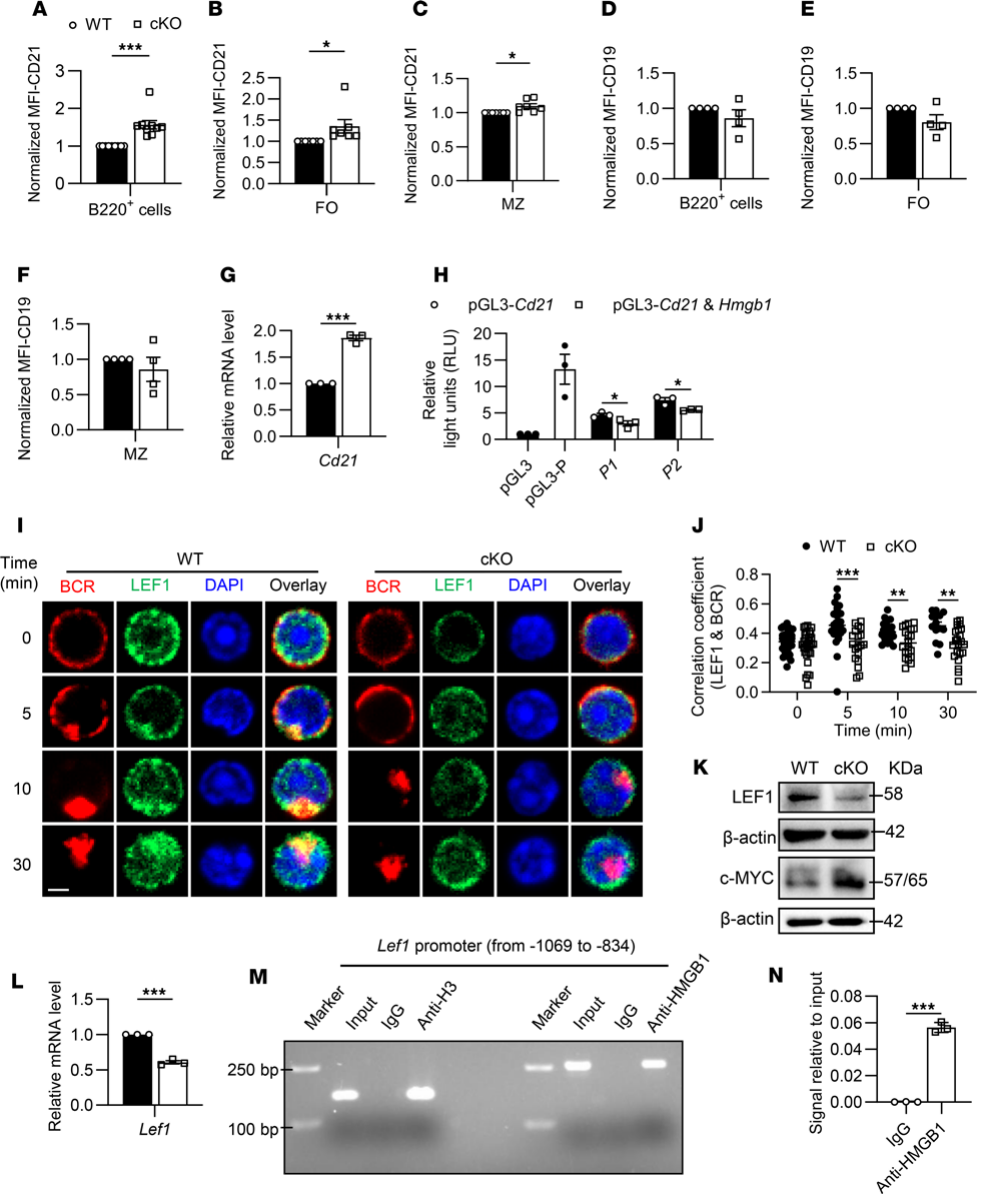
HMGB1 negatively regulates *Cd21* expression to inhibit the B cell activation via binding to *Lef1*. (**A**–**F**) Flow cytometry of CD21 MFI in B220^+^ (*n* = 9), FO (*n* = 7), and MZ B cells (*n* = 9); CD19 (*n* = 4) MFI in B220^+^, FO, and MZ B cells. (**G** and **L**) Quantitative PCR of *Cd21* and *Lef1* mRNA levels in purified splenic B cells (*n* = 3). (**H**) Dual luciferase reporter analysis of *Cd21* promoter activity in HEK293T cells cotransfected with or without *Hmgb1*. (**I** and **J**) Confocal correlation between BCR and LEF1 in splenic B cells stimulated with anti-BCR (60× objective; scale bar = 2.5 μm). Representative confocal microscopy image for 3 independent experiments. (**K**) Western blotting of LEF1 and c-MYC in splenic B cells stimulated with anti-BCR. (**M** and **N**) ChIP-RT-PCR of HMGB1 binding to *Lef1* promoter in purified splenic B cells (*n* = 3). Data are representative of 3 experiments. Two-tailed unpaired Student’s *t* tests (**A**–**G**, **L**, and **N**) and multiple *t* tests (**H** and **J**) were used for statistical analysis. Data shown as mean ± SEM. *P < 0.05, **P < 0.01, ***P < 0.001.

To investigate the potential molecular mechanism by which HMGB1 regulates BCR signal transduction, we assessed expressions of key BCR coreceptors, CD21 and CD19, which lower the activation threshold of BCR. Strikingly, CD21 surface expression was significantly elevated in cKO mice across B220^+^, FO, and MZ B cell subsets, whereas CD19 levels remained unchanged (Figure 8, A–F). Besides, IgM expression increased in B220^+^ and FO B cells, while IgD decreased selectively in FO and MZ cells (Supplemental Figure 5, A–F). Quantitative PCR further verified upregulated *Cd21* mRNA in cKO B cells (Figure 8G), suggesting transcriptional regulation of HMGB1.

To test whether HMGB1 directly suppresses *Cd21* transcription, we conducted luciferase reporter assays in HEK293T cells. Cotransfection of pGL3 vectors containing the *Cd21* promoter (P1/P2) with an *Hmgb1* expression plasmid significantly reduced promoter activity compared with *Cd21* promoter constructs alone (Figure 8H). These results indicate that HMGB1 negatively regulates the *Cd21* promoter activity. While HMGB1 lacks intrinsic transcription factor activity, its DNA-binding capacity and nuclear localization suggest indirect transcriptional modulation.

Building on our prior discovery that LEF1 can repress *Cd21* transcription (11), we observed reduced HMGB1-LEF1 colocalization in cKO B cells (Figure 8, I and J). Intriguingly, both mRNA and protein levels of LEF1 declined in cKO B cells, accompanied by increased expression of c-MYC, which is downstream of LEF1 (Figure 8, K and L). ChIP assays verified direct interaction between HMGB1 and *Lef1* promoter (Figure 8, M and N), implying HMGB1 might regulate *Cd21* promoter through LEF1. Together, these results suggest HMGB1 as a transcriptional coregulator that restrains *Cd21* expression via LEF1, potentially fine-tuning BCR signaling thresholds.

## Discussion

The dual role of HMGB1 as a nuclear DNA chaperone and extracellular DAMP has been extensively studied in innate immunity, yet its intrinsic functions in B cells remained enigmatic. Here we first unveil HMGB1 as a critical rheostat of peripheral B cell homeostasis and activation, operating through integrated transcriptional and cytoskeletal mechanisms. The expansion of MZ B cells and loss of B1a cells in *Hmgb1*-deficient mice highlights HMGB1’s role in peripheral B cell differentiation. These phenotypes align with altered CD21 expression, a key determinant of B cell fate. Prior work established that CD21 levels dictate FO versus MZ differentiation, with elevated CD21 favoring MZ B cell expansion (31, 32), a phenotype recapitulated here through HMGB1 deficiency. Our findings reveal that HMGB1 suppresses *Cd21* transcription potentially by sustaining LEF1 expression via direct binding to the *Lef1* promoter. This finding extends HMGB1’s known regulations in transcription factors such as NF-κB in other cell types, positioning it as a versatile transcriptional coregulator in B cells. However, this mechanism requires further investigation to fully confirm the regulatory role of the HMGB1/LEF1/CD21 axis in B cell development, function, and BCR signaling transduction. A deeper understanding of this axis will help clarify its contribution to the intricate processes governing B cell biology and its potential implications for immune regulation and disease.

Additionally, HMGB1 binds to BCR signalosomes, and the kinetics of its correlation with BCR closely resemble those of distal BCR signaling molecules, such as p-AKT, p-ERK, and p-JNK (33), suggesting that HMGB1 functions as a transducer within BCR signalosomes, modulating downstream signaling events. Moreover, the *Hmgb1*-deficient B cells exhibit the lower activation threshold and overactivated BCR signaling upon anti-BCR stimulation, while the LPS-induced TLR4 signaling was actually impaired. The autophagy and apoptosis of cKO B cells were also altered. Therefore, the B cell phenotype resulting from *Hmgb1* deletion is likely regulated and influenced by a complex interplay of multiple signaling pathways, rather than being determined by a single pathway alone. This multifactorial regulation also helps explain why BCR signaling is enhanced and MZ B cell populations are increased in cKO mice, which seems contradicting to reports showing that MZ B cell development is favored by weaker tonic BCR signaling. Besides, while HMGB1 has been implicated in V(D)J recombination, its dispensability for BM B cell development in this model suggests compartment-specific roles, potentially compensated by redundant factors like HMGB2 during early B cell maturation. These discrepancies highlight the intricate and context-dependent nature of HMGB1’s role in B cell biology, suggesting that its absence may trigger compensatory mechanisms or alternative pathways that reshape B cell behavior in unexpected ways. Further investigation is needed to fully elucidate the molecular networks underlying these phenotypic changes.

Beyond transcriptional regulation, HMGB1 emerges as a modulator of actin dynamics in B cells. The hyperactivation of the MST1/DOCK8/WASP axis in cKO B cells, leading to enhanced F-actin polymerization and BCR clustering, reveals a cytoskeletal role for HMGB1. This is distinct from its reported effects in non-B cells, where HMGB1 regulates actin via moesin phosphorylation or PKA/N-WASP pathways (14, 15). In B cells, HMGB1 constrains actin-driven signalosome assembly by suppressing MST1 activity, thereby limiting sustained BCR microdomain formation. This mechanism aligns with studies emphasizing actin reorganization as a critical checkpoint for B cell activation, where dysregulated cytoskeletal dynamics can lower signaling thresholds and promote autoreactivity. The rescue of BCR hyperactivation by MST1 inhibition further underscores the therapeutic potential of targeting this axis in *Hmgb1*-deficient contexts. Together, the transcriptional (LEF1/CD21) and cytoskeletal mechanism (MST1/DOCK8/WASP) are not isolated but converge to amplify BCR signaling and disrupt homeostasis: Whereas CD21 upregulation lowers the activation threshold and prime B cells for hyperresponsiveness, actin dysregulation enhances signalosome assembly and prolongs signaling duration.

The defective antibody responses in cKO mice following both TI and TD immunizations reveal context-specific roles for HMGB1 in B cell function. The impaired TI immunity despite MZ B cell expansion suggests *Hmgb1* deficiency disrupts the functional maturation or activation thresholds of these cells. Elevated CD21 expression in cKO MZ B cells likely lowers BCR signaling thresholds, promoting premature activation or exhaustion, akin to observations in autoimmune models where hyperactive MZ B cells fail to mount effective TI responses. In contrast, B1a cell loss in cKO mice may directly compromise early IgM production, as B1a cells are primary sources of natural antibodies. This aligns with the reduced serum IgM in cKO mice after TD immunization, where B1a-derived antibodies often prime early pathogen containment. The reduction in GC B cells and class-switched antibodies in immunized cKO mice suggests that HMGB1-mediated signaling restraint is essential for productive immune responses, which also aligns with the impaired humoral immunity in hCD21 transgenic mice (32, 34, 35). Notably, the kidney IgG deposits in cKO mice hint at a breakdown in tolerance, reminiscent of HMGB1’s role in suppressing lupus-like autoimmunity by regulating DNA sensing in B cells. These findings resonate with studies linking HMGB1 to systemic lupus erythematosus pathogenesis, where extracellular HMGB1/DNA complexes drive autoreactive B cell activation (36). However, our work highlights an intracellular, B cell–intrinsic function for HMGB1 in preventing autoantibody production, a dichotomy that warrants further exploration.

The dual mechanisms uncovered here, transcriptional and cytoskeletal, position HMGB1 as a multimodal regulator of B cell function. Its interaction with LEF1 parallels HMGB1’s ability to bend DNA and facilitate transcription factor binding, as seen with NF-κB in innate immune cells. Similarly, its cytoskeletal role via MST1/DOCK8/WASP expands upon HMGB1’s known capacity to modulate cell migration in cancer and endothelial cells (37, 38). The convergence of these pathways in regulating BCR signaling underscores HMGB1’s evolutionary versatility as a mediator of immune cell behavior. The autoimmune features in *Hmgb1*-cKO mice suggest that HMGB1 dysregulation could contribute to B cell–driven pathologies. Conversely, HMGB1 inhibition might enhance BCR signaling in contexts requiring amplified immunity, such as vaccines or infections. However, the balance between beneficial and detrimental effects will require careful dissection, particularly given HMGB1’s extracellular proinflammatory roles. Future studies should explore HMGB1’s interactions with TLR2/CD36 in B cells, its role in human autoimmune disorders, and whether its cytoskeletal functions intersect with metabolic pathways like oxidative phosphorylation, which was impaired in cKO B cells.

In conclusion, our study redefines HMGB1 as a guardian of B cell homeostasis, integrating nuclear and cytoplasmic pathways to modulate activation thresholds. By bridging transcriptional control and cytoskeletal dynamics, HMGB1 ensures that B cells remain poised for defense without tipping into autoimmunity, a delicate equilibrium essential for immune health.

## Methods

### Sex as a biological variable.

Male and female mice aged 6–8 weeks were used in a 1:1 ratio. Sex was not considered as a biological variable in these analyses.

### Mice and cells.

*Hmgb1*-cKO mice on the C57BL/6 background were obtained by crossing *Cd19*-Cre mice from Jackson Laboratory with *Hmgb1*^fl/fl^ mice from Cyagen Biosciences. All mice were maintained under specific pathogen–free conditions.

Splenic lymphocytes were isolated by Ficoll-Paque Premium (17-5446-02; GE Healthcare, now Cytiva). Then splenic B cells were obtained with anti-Thy1.2 mAb (105310; BioLegend) and guinea pig complement (C300-0500; Rockland Immunochemicals). HEK293T cells were provided by Hongmei Yang (Huazhong University of Science and Technology).

### Flow cytometry.

Single-cell suspensions from BM were lysed with red cell lysis buffer (RT122-02; TIANGEN), filtered, and then stained with the anti-mouse antibodies, including PE-anti-Ly-51 (108307; BioLegend), APC-anti-CD43 (143208; BioLegend), PE/Cy7anti-CD24 (101822; BioLegend), BV421-anti-IgM (406518; BioLegend), and BV510-anti-B220 (103247; BioLegend). Splenic cells were stained with the following specific anti-mouse antibodies: FITC-anti-CD95 (152606; BioLegend), Percp-anti-B220 (103234; BioLegend), AF647-anti-GL7 (144606; BioLegend), FITC-anti-CD19 (101506; BioLegend), PE-anti-CD23 (101608; BioLegend), Percp/Cy5.5-anti-IgD (405710; BioLegend), APC-anti-CD21 (123412; BioLegend), BV421-anti-IgM, BV510-anti-B220, FITC-anti–Annexin V (640906; BioLegend), and FITC-anti-B220 (103206; BioLegend). For intracellular molecule staining, PE/Cy7-anti-KI67 (25-5698-82; eBioscience) was labeled after cells were fixed and permeabilized using Fixation/Permeabilization Kit (00-5123, 00-5223; eBioscience). Peritoneal cells were flushed from the abdominal cavity and stained with these antibodies: FITC-anti-CD19 (101506; BioLegend), Percp/Cy5.5-anti-IgD (405710; BioLegend), PE/Cy7-anti-CD5 (100622; BioLegend), BV421-anti-IgM (406518; BioLegend), and APC/Cy7-anti-CD11b (101226; BioLegend). For the immunization experiment, PE-anti-NP (N-5070-1; Biosearch Technologies), BV421-anti-IgD, and BV510-anti-CD138 (142521; BioLegend) were used for staining to assess PC, PBC, and MBC subsets. Cells were incubated at 4°C and detected with Attune NxT flow cytometer (Thermo Fisher Scientific) and analyzed with FlowJo V10 (Tree Star) software.

### Immunofluorescence.

Mice’s kidneys were cross-sectioned and the largest cross sections were embedded in OCT (4583; Sakura Finetek) and frozen in liquid nitrogen to prepare frozen sections. Sections were blocked and stained with Cy3-AffiniPure Donkey Anti-Mouse IgG (715-165-151; Jackson ImmunoResearch), imaged using a confocal microscope (LSM 780; Zeiss), and finally analyzed using the ZEN 2.3 software. To identify B cells and the MZ region, spleen sections were stained with Rat anti-Mouse CD169 (MCA947GA; Bio-Rad), followed by Goat anti-Rat IgG (H+L) Cross-Adsorbed Secondary Antibody, Alexa Fluor 488 (A-11006; Thermo Fisher Scientific), to visualize the MZ; after 2 washes, spleen sections were stained with APC-anti-B220 (553092; BD Pharmingen) to demonstrate the B cells. Images were captured via the Nikon confocal microscope and analyzed using NIS-Elements AR 3.2 software.

### Confocal analysis.

For confocal analysis, purified splenic B cells were immobilized on glass slides coated with poly-l-lysine (12-545-F; Fisherbrand) and then incubated on ice, as soluble antigen, with Alexa Fluor 594-(Fab′)_2_-anti-Ig(M+G) (115-586-068; Jackson ImmunoResearch) for half an hour and with streptavidin (16000114; Jackson ImmunoResearch) for 10 minutes to activate B cells. Cells were then stimulated for 5, 10, or 30 minutes at 37°C. After each time point, cells including those in the resting stage were fixed with 4% paraformaldehyde (18908; Thermo Fisher Scientific) and permeabilized with 0.05% saponin (S4521-10G; Sigma). Cells were labeled with the following specific antibodies: anti-HMGB1 (ab190377; Abcam); anti-pCD19 (ab203615; Abcam), anti-Phosphotyrosine (05-321; Merck), anti-pBTK (ab52192; Abcam), anti-pSHIP-1 (3941S; Cell Signaling Technology [CST]), anti-pWASP (A300-205A; Bethyl Laboratories), AF488-phalloidin (R37110; Thermo Fisher Scientific), anti-DOCK8 (sc292124; Santa Cruz Biotechnology), anti-pNF-κB-pP65 (3033S; CST), anti-pSTAT1 (9167S; CST), anti-pSTAT5 (4322S; CST), and anti-LEF1 (14972-1-AP; Proteintech). Images were captured using the Nikon TIRFm system (Nikon Eclipse Ti-PFS) with 405, 488, 546, and 647 nm lasers, and colocalization and MFI were determined with the NIS-Elements AR 3.2 software.

### Western blotting.

Splenocytes were incubated with or without biotin-conjugated F(ab′)_2_ Ig (M+G) (115-066-068; Jackson ImmunoResearch) for 30 minutes and streptavidin (16000114; Jackson ImmunoResearch) for 10 minutes on ice, then stimulated for 5, 10, and 30 minutes at 37°C, except for those in the resting stage. Cells were then lysed using RIPA lysis buffer (Beyotime; P0013B) containing protease inhibitor cocktail (1×) (G2006; Servicebio), 10 mM NaF (G2007-1; Servicebio), and 1 mM Na_3_VO_3_ (G2007-2; Servicebio). SDS-PAGE loading buffer (5×) (BL502A; Biosharp) was added to the cell lysates, and proteins were separated using SDS-PAGE gel and transferred to a nitrocellulose membrane for detection using the following antibodies: anti-pCD19 (3571S; CST), anti-CD19 (90176S; CST), anti-pY, anti-pBTK (5082S; CST), anti-BTK (8547S; CST), anti-pSHIP-1 (3941S; CST), anti-SHIP-1 (2728S; CST), anti-pPI3K (4228S; CST), anti-PI3K (4292S; CST), anti-pAKT (4060S; CST), anti-AKT (9272S; CST), anti-pFOXO1 (9461S; CST), anti-FOXO1 (2880S; CST), anti-pS6 (4856S; CST), anti-S6 (2217S; CST), anti-pmTOR (5536S; CST), anti-mTOR (5536S; CST), anti-pWASP (ab59278; Abcam), anti-WASP (sc13139; Santa Cruz Biotechnology), anti-pEzrin (3726S; CST), anti-WIP (sc271113; Santa Cruz Biotechnology), anti-DOCK8 (sc292124; Santa Cruz Biotechnology), anti-pMST1 (3681S; CST), anti-MST1 (PA5-22015; Thermo Fisher Scientific), anti-pIKKα/β (2697S; CST), anti-IKKα/β (8943S; CST), anti-pNF-κB-pP65 (3033S; CST), anti-NF-κB-P65 (4764S; CST), anti-pSTAT1 (9167S; CST), anti-STAT1 (14994; CST), anti-pSTAT5 (4322S; CST), anti-STAT5 (ab194898; Abcam), anti-LEF1 (2286S; CST), anti-c-Myc (13987S; CST), anti-HMGB1 (ab190377; Abcam), pJNK1/2 (AP0473; Abclonal), LC3 (A5618; Abclonal), AID (A16217; Abclonal), Caspase-3 (A19654; Abclonal), anti-GAPDH (A19056; Abclonal), Hsp90 (A5027; Abclonal), and Beta-Actin (60008-1-lg; Proteintech).

### Seahorse assay.

Twenty-four–well microplates were treated with 50 μg/mL of poly-d-lysine (C0132; Beyotime) overnight at 4°C. Purified splenic B cells (2 × 10^6^) were stimulated with 10 μg/mL LPS or 10 μg/mL F(ab′)_2_ Ig (M+G) for 2 hours, then transferred into the Seahorse 24-well microplates, where cells were washed 3 times with Seahorse Assay Medium containing 25 mM glucose (G8769; Sigma), 2 mM l-glutamine (G6392; Sigma), and 1 mM sodium pyruvate (S8636; Sigma). Under the basic conditions, the OCR was then measured using XF24 Extracellular Flux Analyzer (Seahorse Bioscience) by the reaction of 1.5 μM oligomycin (abs42024304; Absin), 1 μM FCCP (C2920; Sigma), and 0.5 μM rotenone (R8875; Sigma) with the addition of 1 μM antimycin A (abs42013402; Absin).

### Calcium flux assay.

Purified B cells (5 × 10^5^) were incubated with 1 μM Fluo-4AM (S1060; Beyotime) in a 96-well plate for half an hour, following which, the cells were washed 3 times and mixed with F(ab′)_2_ Ig (M+G). The relative intracellular calcium levels were measured using flow cytometry according to Fluo-4AM manufacturer’s protocol.

### B cell proliferation assay.

Purified B cells (5 × 10^5^) were labeled with CTV (C34557; Thermo Fisher Scientific) and stimulated with 5 μg/mL LPS (L2880; Sigma) or 10 μg/mL CpG (tlrl-1826-1; InvivoGen). After 72 hours, cells were incubated with FITC-anti-B220 (103206; BioLegend) and Percp-anti-7-AAD (559925; BD Pharmingen) on ice, then detected using Attune NxT flow cytometer (Thermo Fisher Scientific). Data were analyzed using the FlowJo V10 (Tree Star) software.

### Phos flow.

Splenocytes (2 × 10^6^) were incubated with Percp-anti-B220 and biotin-conjugated F(ab′)_2_ Ig (M+G) for half an hour on ice, then mixed with streptavidin for 10 minutes. After activation for 5, 10, and 30 minutes at 37°C, cells were fixed with Phosflow Lyse/Fix buffer and permeabilized with Phosflow Perm buffer III (BD Biosciences). Cells were stained with anti-pWASP (ab59278; Abcam) and AF488-phalloidin (R37110; Thermo Fisher Scientific) and detected using Attune NxT flow cytometer. The data were analyzed using FlowJo V10 software.

### Treatment with rapamycin and XUM-MP-1 inhibitor in vitro.

Purified B cells were treated with 10 μM XUM-MP-1 (T4212; TargetMol) or 20 nM rapamycin (HY-10219; MedChemExpress) at 37°C for 2 hours before the first step of the Western blotting experiment.

### TIRFm.

For TIRFm analysis, splenocytes were incubated with Alexa Fluor 594-mB-Fab’-anti-Ig (115-587-003; Jackson ImmunoResearch) and activated by biotin-conjugated F(ab’)_2_ Ig (M+G) (115-066-068; Jackson ImmunoResearch) tethered lipid bilayer prepared as previously described (39). After being fixed and permeabilized, cells were stained with specific antibodies, including AF488-phalloidin (R37110; Thermo Fisher Scientific), anti-pWASP (A300-205A; Bethyl Laboratories), anti-pBTK, anti-pY, anti-pSHIP-1, and anti-pCD19. Images were obtained using the TIRFm system (Eclipse Ti-PFS; Nikon) and analyzed using NIS-Elements AR 3.2 software. The B cell contact area was determined using IRM images. Background fluorescence caused by Ag tethering to lipid bilayers was subtracted in the absence of B cells or secondary Ab controls. Each set of data was obtained from more than 50 individual cells pooled from 3 independent experiments. F-actin foci were calculated using the “Analyze Particles” plugin of ImageJ, from 20 individual cells pooled from 3 independent experiments.

### Immunization and ELISA.

For NP-KLH immunization, NP-KLH (MPL+TDM; N-5060-25; Biosearch Technologies) was mixed with an adjuvant (MPL+TDM; S6322-1VL, Sigma) to form an NP-KLH/adjuvant mixture. WT and *Hmgb1*-cKO mice aged 6–8 weeks were intraperitoneally injected with 40 μg NP-KLH/adjuvant on the first day. On day 14, the serum samples were collected, and on day 28, mice were intraperitoneally injected with 40 μg of NP-KLH/adjuvant for the secondary immunization. On day 33, the serum samples were obtained again, splenic cells were lysed to remove erythrocyte, and splenocytes were stained with the anti-mouse antibodies for flow cytometry analysis. The collected serum samples were analyzed using ELISA, whereby 200 μg/mL NP-bovine serum albumin (N-5050H-100; Biosearch Technologies) was used for coating plates, while IgM (A90-101P; Bethyl Laboratories) and IgG1 (A90-105P; Bethyl Laboratories) were used as specific secondary antigens to detect the antibody levels of the NP-specific subgroups. Absorbance at 450 nm was measured using the microplate reader Tecan M200 Pro.

### Luciferase reporter assay.

TranslT-293 Transfection Reagent (MIR2700; Mirus) was used to cotransfect pGL3-*Cd21* (P1 or P2) alone or with pGl3-*Hmgb1* and the internal control plasmid-pRL-TK into HEK293T cells in 24-well microplates. The luciferase reporter assay was performed based on the instructions of the Dual Luciferase Reporter Gene Assay Kit (RG027; Beyotime): *Cd21* P1: –890 to +105; *Cd21* P2: –1,982 to +105 (Figure 8H).

### Quantitative RT-PCR analysis.

Purified B cells (5 × 10^6^) were used to extract total RNA according to AxyPrep Multisource RNA Kit (AP-MN-MS-RNA-50; Axygen). cDNA was obtained using PrimeScript RT Reagent Kit with gDNA Eraser (RR047A; Takara), and the expression of different genes was measured by the StepOne Real-Time PCR System (Allied Biosystems) using SYBR Premix ex taq (RR047A; Takara). Sequences of the primer sets used are *Hmgb1* promoter forward 5′-AGGGCACCCCAACTTTTCAC-3′, reverse 5′-TAGCAGACATGGTCTTCCACC-3′; *Cd21* promoter forward 5′-GCAAAACTGTCTGGTGCCAG-3′, reverse 5′-TATGTCACAGACAACCCCGC-3′; *Lef1* promoter forward 5′-TGAGTGCACGCTAAAGGAGA-3′, reverse 5′-CTGACCAGCCTGGATAAAGC-3′.

### ChIP assay.

ChIP experiments were conducted based on the manufacturer’s protocol by using the SimpleChIP Enzymatic Chromatin IP Kit (Agarose Beads) (9002S; CST). Purified B cells from WT mice were transferred into formaldehyde for 10 minutes, and then ultrasound was used on the lysate to shear cross-linked chromatin into lengths ranging from 100 to 1,000 base pairs. Solubilized chromatin was immunoprecipitated using anti-HMGB1, and PCR assay was conducted on a Bio-Rad PCR instrument. ChIP DNA was quantified using a StepOne Real-Time PCR System (Allied Biosystems) instrument. The free and purified DNA was normalized to the input material used for the ChIP reaction.

Sequences of the primer sets used for PCR and quantitative RT-PCR are as follows: *Mst1* promoter forward 5′-ATGGTGGCAGGAAGACAGTG-3′, reverse 5′-GCCTGGACTTGACTGACACA-3′; *WIP* promoter forward 5′-AGTGTCCCAACAGAAGGGTC-3′, reverse 5′-TGTACTGCCCAAAGGTTCGC-3′; *Dock8* promoter forward 5′-CTCGTGGGATGCAGCCATTA-3′, reverse 5′-TGGATCCAGAAGCAGCCAG-3′; *Lef1* promoter forward 5′-CCTGGCATTCGGACATTCCC-3′, reverse 5′-CAAATGTTCCAGCCCGCAG-3′.

### RNA extraction and RNA-Seq analysis.

For RNA preparation, Biotin Mouse B Lymphocyte Enrichment Cocktail (51-9001846; BD Biosciences) and Streptavidin Particles Plus–DM (51-9000810; BD Biosciences) of Mouse B Lymphocyte Enrichment Set-DM Kit (557792; BD Biosciences) were used to obtained purified B lymphocytes from splenic lymphocytes of WT and cKO mice according to the manufacturer’s protocol. Cells were treated with TRIzol reagent (15596026; Thermo Fisher Scientific) for RNA extraction and then sequenced using the BGIseq-500 system (BGI). PolyA tail mRNA was enriched with magnetic beads containing Oligodt. The RNA obtained was fragmented using a disruption buffer, followed by reverse transcription with random N6 primers; subsequently, the 2 cDNA strands were synthesized to form double-stranded DNA. The ends of the synthesized double-stranded DNA were blunted, and their 5′-ends were phosphorylated. Ligation products were then amplified via PCR with specific primers. After the PCR product was thermally denatured into single strands, these single-stranded DNAs were cycled using a bridging primer to generate a single-stranded circular DNA library. WT and *Hmgb1*-cKO B cell genome, which contains 15,410 genes, was filtered to obtain clean reads by using SOAPnuke software (BGI). The clean data were aligned using HISAT and bowtie2 and used to calculate the expression level of each gene by using RNA-Seq with expectation maximization. Finally, biological pathway classification and enrichment analysis of the differential genes were conducted to calculate the *P* value, following which the FDR correction was performed. *Q* value of ≤ 0.05 was considered significant enrichment.

### H&E staining.

Kidney, colon, lung, and liver of mice were placed in 4% paraformaldehyde (diluted with PBS) and fixed at room temperature. After 48 hours, the tissues were removed for dehydration and embedded with paraffin; slices of 4 μm thickness were obtained using a paraffin slicer. Following deparaffinization, the slides were stained with H&E, and images were obtained using an optical microscope.

### Apoptosis detection.

The purified splenic B cells (3 × 10^5^) were stimulated with 10 μg/mL LPS or 3 μg/mL F(ab′)_2_ Ig (M+G) at 37°C for 24 hours. Then cells were stained with BV510-anti-B220 at 4°C for 30 minutes, washed twice, and incubated with Annexin V-FITC and PI staining solution (A211; Vazyme) at room temperature for 10 minutes. Finally, cells were detected with Attune NxT flow cytometer.

### Transwell cell migration assay.

Purified B cells with equal number (1 × 10^6^) from WT and cKO mice were resuspended in culture medium and seeded into the upper chamber of a 24-well Transwell insert (725201; NEST). The lower chamber was filled with medium containing 100 ng/mL CXCL12 (250-20B; PeproTech) as a chemoattractant (the blank control group received PBS instead of CXCL12). The plates were then incubated at 37°C in a 5% CO_2_ humidified atmosphere for 16 hours. After incubation, the Transwell inserts were carefully removed, and the cells that had migrated to the lower chamber were collected and counted. The migration efficiency was calculated as the percentage of migrated cells relative to the total number of cells initially seeded in the upper chamber.

### V(D)J recombination examination.

Genomic DNA was extracted from sorted BM pre-B (B220^+^IgM^–^CD43^–^) cells and pro-B (B220^+^IgM^–^CD43^+^) cells. Degenerate PCR primers for detection of genomic recombination across the proximal V_H_7183 and V_H_Q52 regions to J3 segments, and Vκ region to Mar35 segment were used as described (40, 41): VH7183 (5′-CGGTACCAAGAASAMCCTGTWCCTGCAAATGASC-3′), VHQ52 (5′-CGGTACCAGACTGARCATCASCAAGGACAAYTCC-3′), JH3 (5′-GTCTAGATTCTCACAAGAGTCCGATAGACCCTGG-3′), VκD (5′-GGCTGCAGSTTCAGTGGCAGTGGRTCWGGRAC-3′), and Mar35 (5′-AACACTGGATAAAGCAGTTTATGCCCTTTC-3′). The constant region was conducted by using primers Cμ 5′ (5′- TGGCCATGGGCTGCCTAGCCCGGGACTT-3′) and Cμ 3′ (5′-GCCTGACTGAGCTCACACAAGGAGGA-3′). PCR products were separated using agarose gel electrophoresis and observed by ethidium bromide staining.

### Statistics.

Data represent results from a minimum of 3 independent experiments. Prism 10.1.2 (GraphPad software) was used to perform 2-tailed unpaired Student’s *t* tests, multiple 2-tailed *t* tests, and 1-way ANOVA to evaluate the statistical significance. *P* < 0.05 was considered statistically significant.

### Study approval.

All animal experiments were conducted in strict compliance with the Chinese Council on Animal Care guidelines. The experimental protocols were reviewed and approved by the Ethics Committee of Animal Experimentation of Tongji Medical College, Huazhong University of Science and Technology (Wuhan, China).

### Data availability.

Raw sequencing data are available under National Center for Biotechnology Information BioProject accession PRJNA1243072.

## Author contributions

Qiuyue Chen, ZZ, and CL drafted up the manuscript. Qiuyue Chen, ZZ, and NX analyzed the data and generated figures. CL designed research and reviewed and revised the manuscript. Qiuyue Chen, ZZ, NX, LL, and XD performed the ELISA, TIRFm experiments, ChIP, and quantitative RT-PCR analysis. Qiuyue Chen, YJ, and DK carried out the H&E staining, RNA extraction, and RNA-Seq analysis. LY, Qiuyue Chen, NL, PJ, and XD carried out the luciferase reporter assay and Seahorse experiments. Qiuyue Chen, ZZ, NX, LY, YZ, and JC performed the immunofluorescence and confocal experiments. Qiuyue Chen, ZZ, NX, LL, XD, YJ, LY, and DK performed the flow cytometry assay. ZZ, NL, PJ, YZ, JC, JL, and YH carried out Western blotting. ZZ, QG, NX, Qianglin Chen, and PJ performed the calcium flux, B cell proliferation assay, and phos flow. NX, HM, XZ, and FZ assisted with manuscript preparation and revision and provided helpful comments on the content.

## Supplementary Material

Supplemental data

Unedited blot and gel images

Supporting data values

## Version 1 09/23/2025

Electronic publication
