# Supplementary material for: HMGB1 couples LEF1 to regulate B cell immunity
Source: JCI Insight. 2025 Sep 23;10(18):e187002. doi: 10.1172/jci.insight.187002 (PMC12487846; doi:10.1172/jci.insight.187002)
Supplement: Supplemental data [file jciinsight-10-187002-s188.pdf]

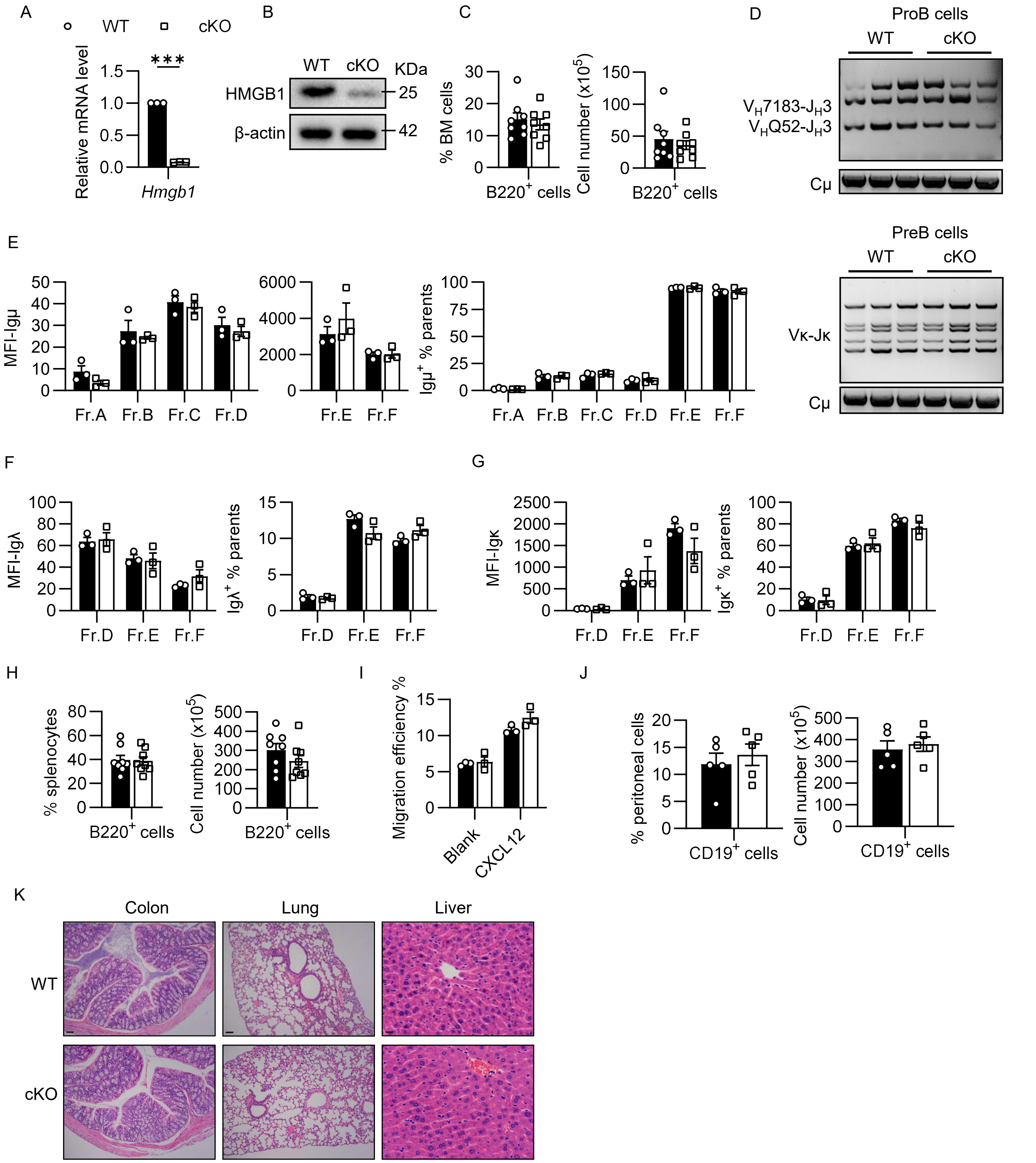

Fig. S1. HMGB1 is dispensable for maintaining total B cell numbers in the bone marrow, spleen, and peritoneal cavity, as well as for V(D)J recombination in B cells. (**A**) Quantitative PCR analysis of *Hmgb1* mRNA levels in purified splenic B cells from WT and cKO mice (n = 3). (**B**) Western blot analysis of HMGB1 protein expression in splenic B cells. (**C**, **H** and **J**) Mean proportions and absolute numbers of B220$^+$ cells in bone marrow, spleen, and CD19$^+$ cells in the peritoneal cavity. (**D**) Semi-quantitative PCR analysis of V_H and Vκ chain rearrangements by degenerate primers in sorted bone marrow pro-B (B220$^+$IgM$^-$CD43$^+$) and pre-B (B220$^+$IgM$^-$CD43$^-$) cells (n = 3). (**E**-**G**). Flow cytometry analysis of Igμ, Igκ and Igλ MFI, as well as Igμ$^+$, Igκ$^+$ and Igλ$^+$ populations, in bone marrow B cell subsets (n = 3) (**I**) Migration efficiency of splenic B cells toward CXCL12, assessed by Transwell assay (n = 3). (**K**) Representative H&E-stained sections of colon, lung, and liver (colon/lung: 10× objective, scale bar = 200 μm; liver: 40× objective, scale bar = 50 μm). Shown are representative images of H&E staining from one of three independent experiments. Data are representative of three independent experiments. Two-tailed unpaired Student's t-tests (**A**, **C**, **H**-**J**) and multiple t-tests (**E**-**G**) were used for statistical analysis. Data shown as mean ± SEM. *** p<0.001.

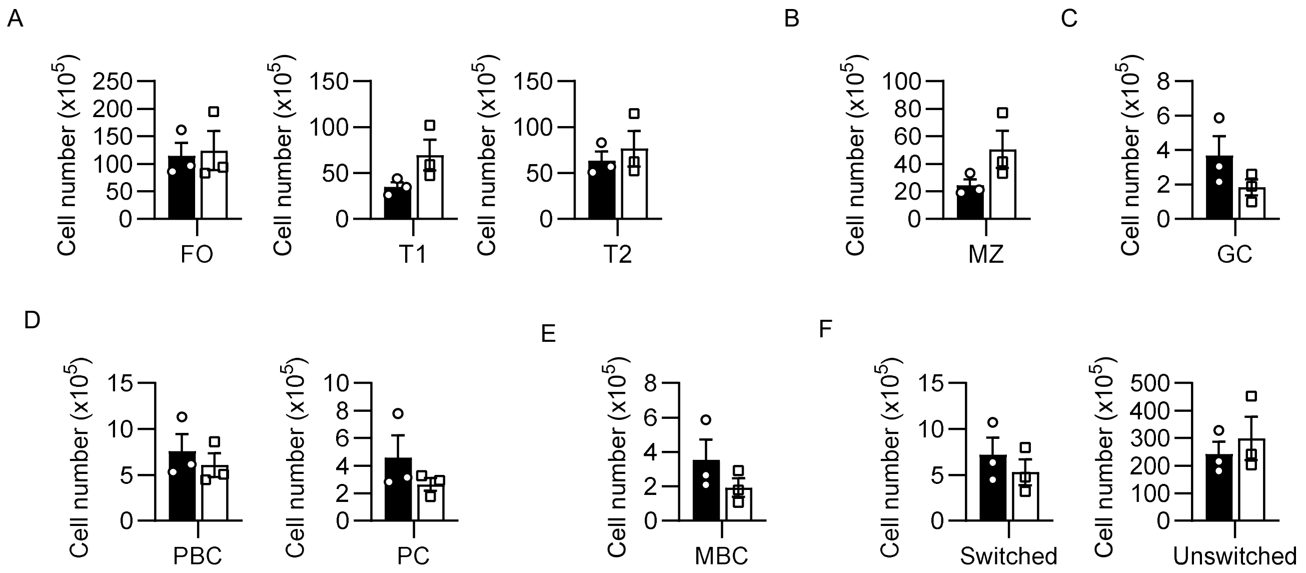

Fig. S2. The numbers of splenic cell subsets in cKO mice remains unchanged after being immunized by NP-Ficoll. (**A-F**) Absolute numbers of splenic B cell subpopulations in WT (n = 3) and cKO (n = 3) mice immunized with NP-Ficoll, analyzed by flow cytometry. Data are representative of three independent experiments. Two-tailed unpaired Student's t-tests were used for statistical analysis. Data shown as mean ± SEM. * $p<0.05$, ** $p<0.01$, *** $p<0.001$.

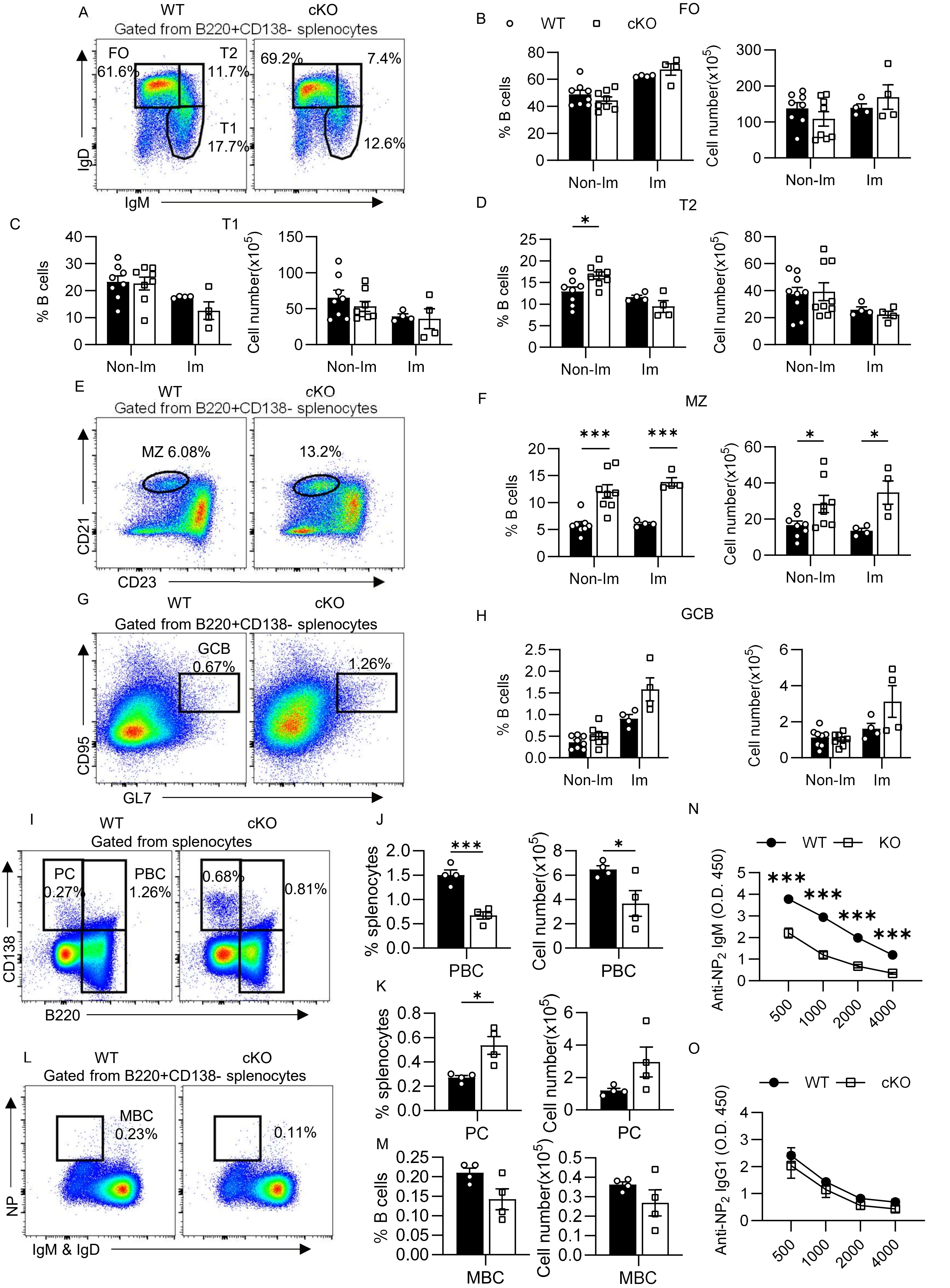

Fig. S3. HMGB1 participates in the primary TD-immune response. (**A**-**M**) Flow cytometry analysis of splenic cell subpopulations in WT (n = 4) and cKO (n = 4) mice 14 days after primary NP-KLH immunization. Mean proportions and absolute numbers are shown. (**N-O**) ELISA quantification of serum NP-IgM and NP-IgG1 titers (OD values) in primarily NP-KLH immunized mice. Data are representative of three experiments. Multiple t-tests (**B-D**, **F**, **H**, **N-O**) and two-tailed unpaired Student's t-tests (**J-K**, **M**) were used for statistical analysis. Data shown as mean ± SEM. * p<0.05, ** p<0.01, *** p<0.001.

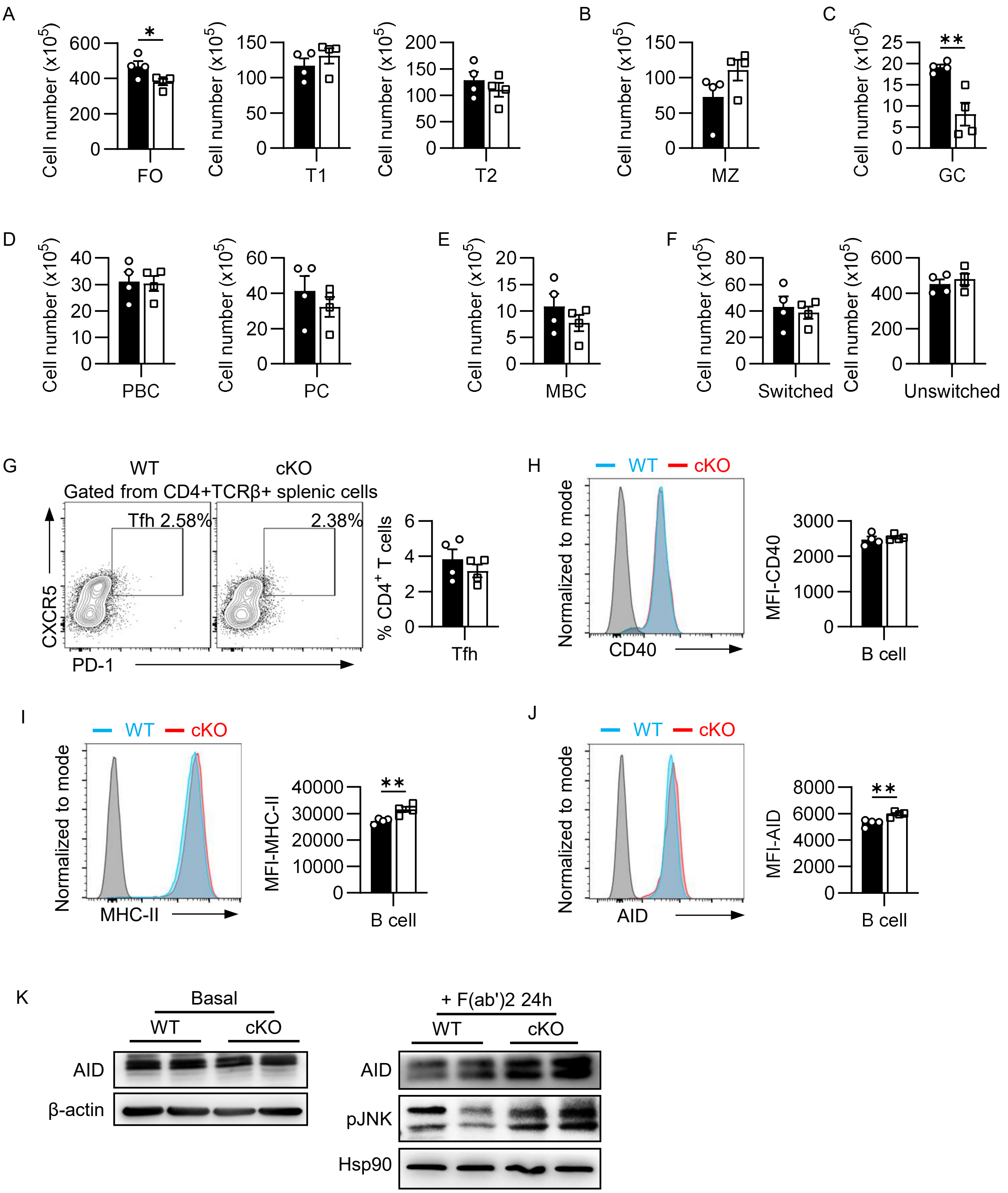

Fig S4. Secondary TD-immune response and signaling were altered in *Hmgb1* cKO mice. (**A-F**) Flow cytometry analysis of splenic subpopulation numbers in secondarily NP-KLH-immunized mice (n = 4). (**G**) Flow cytometry analysis for the proportion of Tfh cells. (**H**) Flow cytometry analysis for MFI of CD40, MHC II, and AID in B cells from secondarily NP-KLH-immunized mice. (**K**) Western blot analysis of AID expression in resting B cells, AID and pJNK1/2 expressions in B cells stimulated by anti-BCR for 24h. Data are representative of three experiments. Two-tailed unpaired Student's t-tests were used for statistical analysis. Data shown as mean ± SEM. * $p<0.05$, ** $p<0.01$.

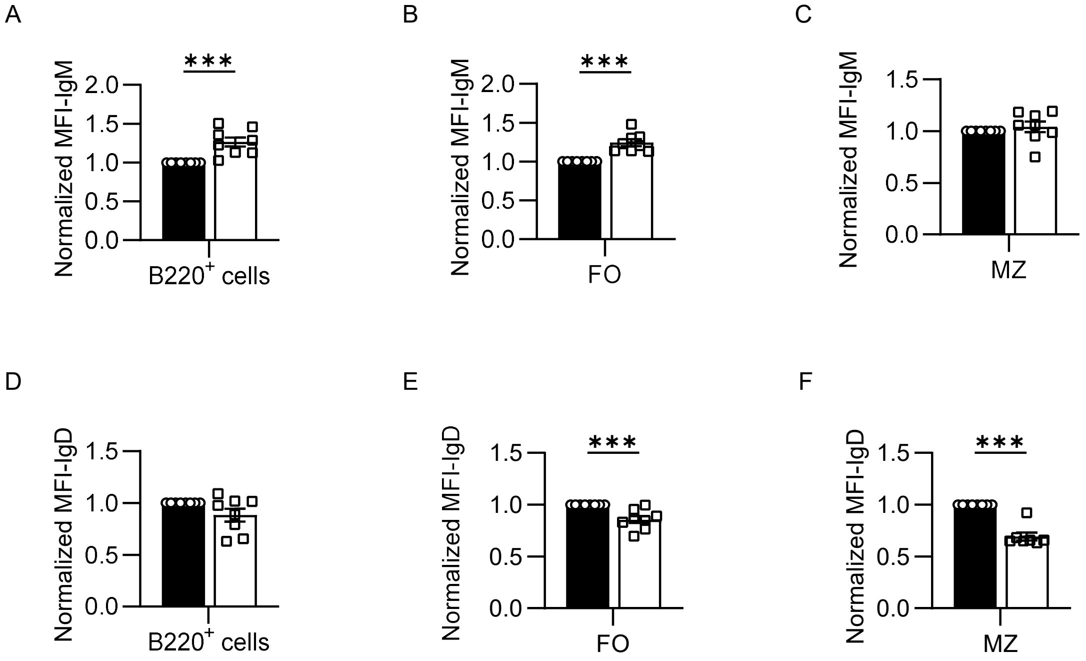

Fig. S5. Abnormal expressions of surface IgM and IgD in cKO B cell subsets. (**A**-**C**) Flow cytometry analysis of IgM MFI (n = 8) in B220^{+}, FO, and MZ B cells. (**D**-**F**) IgD MFI in B220^{+} (n = 9), FO (n = 8), and MZ B cells (n = 7). Data are representative of three independent experiments. Two-tailed unpaired Student's t-tests were used for statistical analysis. Data shown as mean ± SEM. *** $p<0.001$.

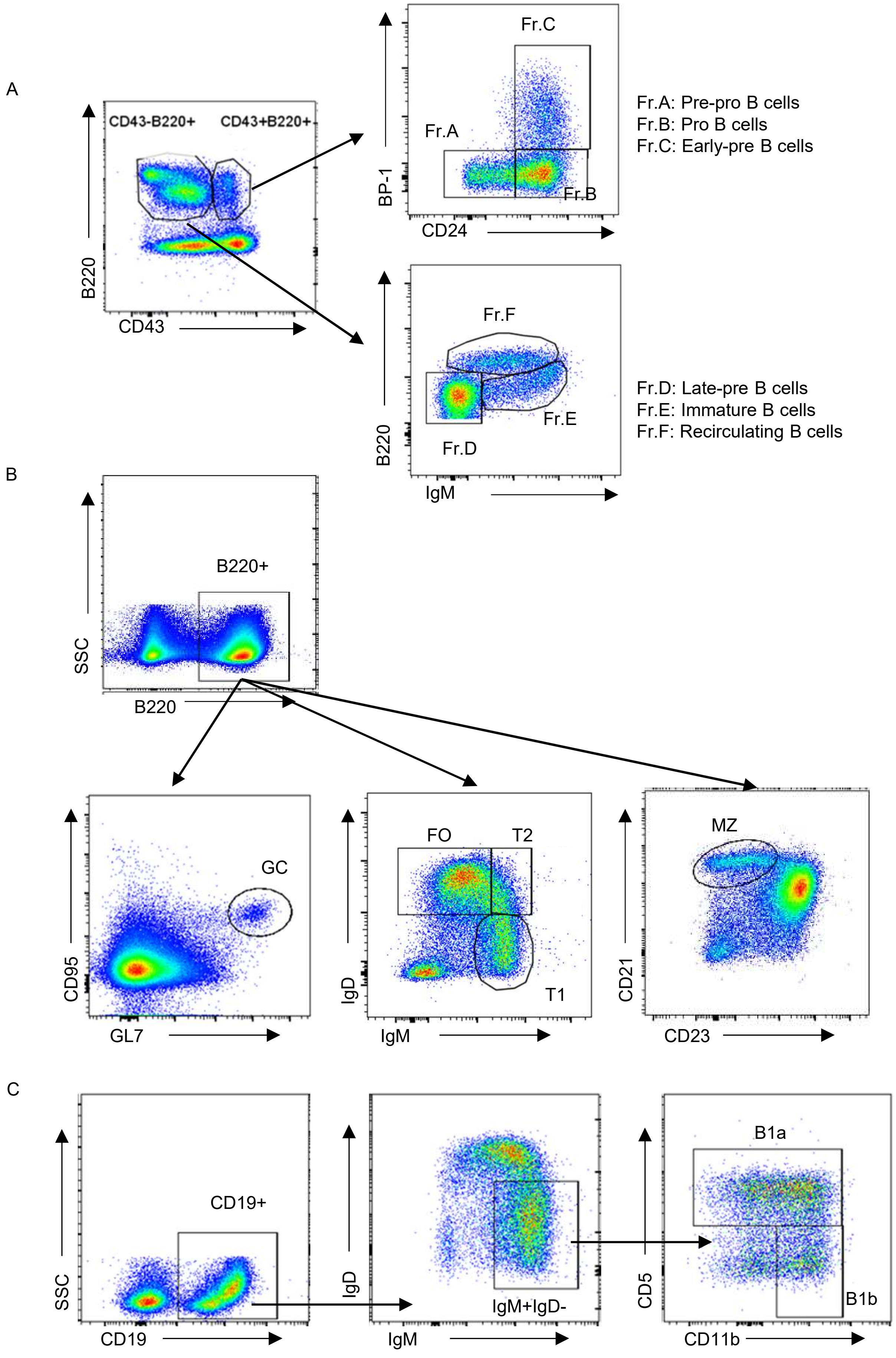

Fig. S6. Flow cytometry gating strategies. Gating schemes for: **(A)** bone marrow cells, **(B)** splenic lymphocytes, and **(C)** peritoneal cavity cells.
