# Supplementary material for: HMGB1 couples LEF1 to regulate B cell immunity
Source: JCI Insight. 2025 Sep 23;10(18):e187002. doi: 10.1172/jci.insight.187002 (PMC12487846; doi:10.1172/jci.insight.187002)
Supplement: Unedited blot and gel images [file jciinsight-10-187002-s189.pdf]

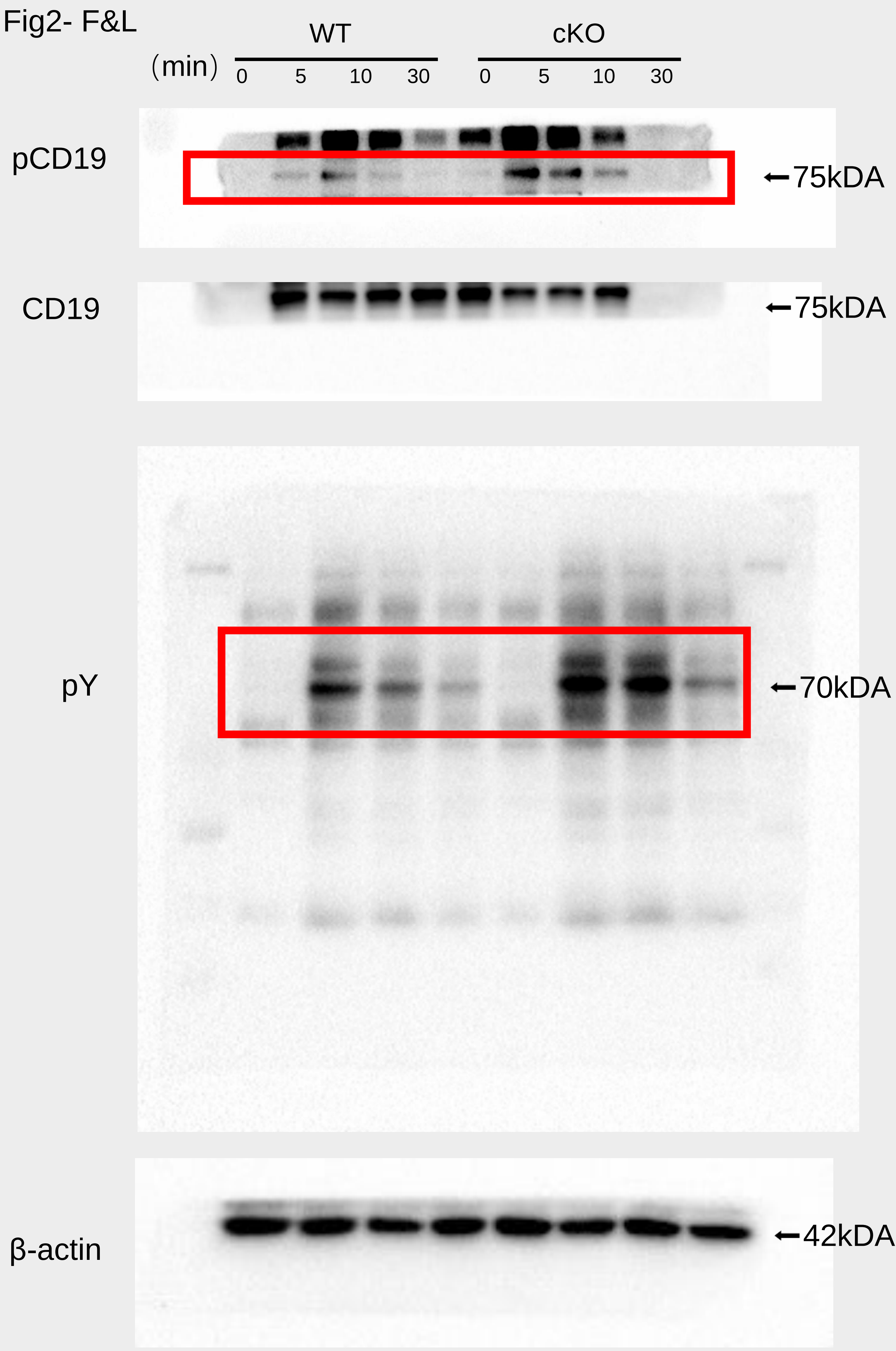
Fig2- F&L
WT
cKO
(min)
0
5
10
30
0
5
10
30
pCD19
←75kDA
CD19
←75kDA
pY
←70kDA
β-actin
←42kDA

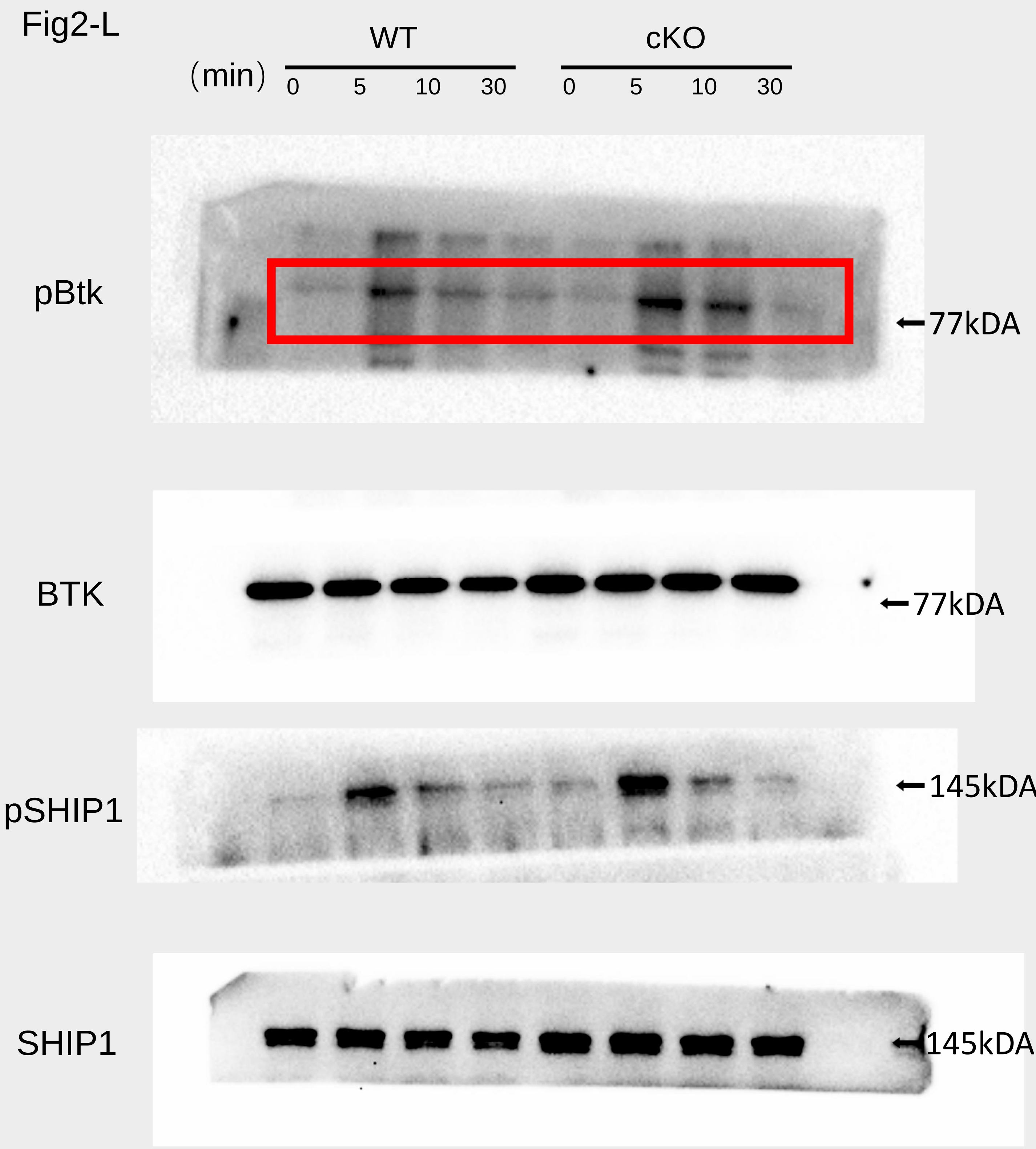
Fig2-L
WT
cKO
(min)
0
5
10
30
0
5
10
30
pBtk
77kDA
BTK
77kDA
pSHIP1
145kDA
SHIP1
145kDA

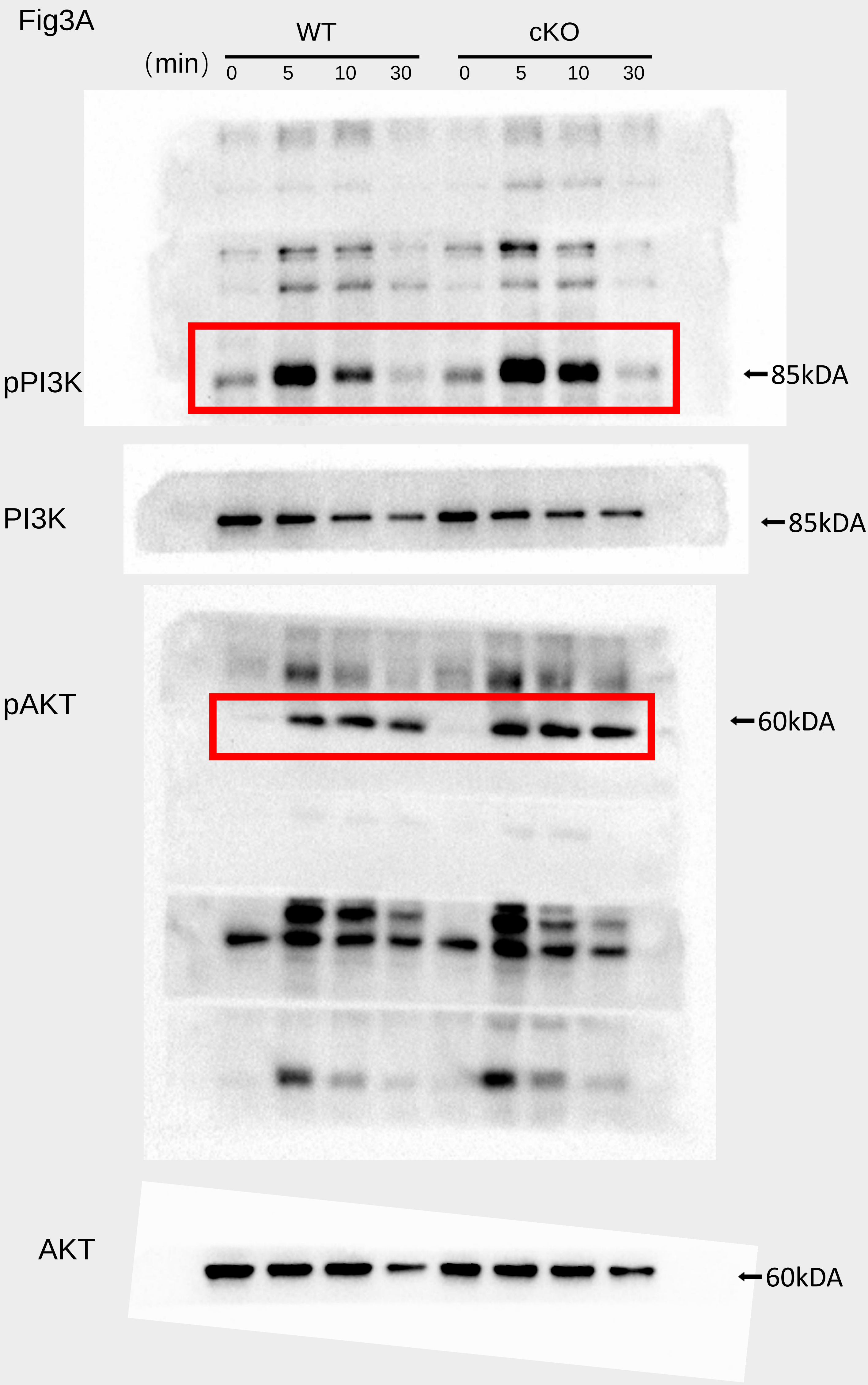
Fig3A
WT
cKO
(min)
0
5
10
30
0
5
10
30
pPI3K
85kDA
PI3K
85kDA
pAKT
60kDA
AKT
60kDA

Fig3A

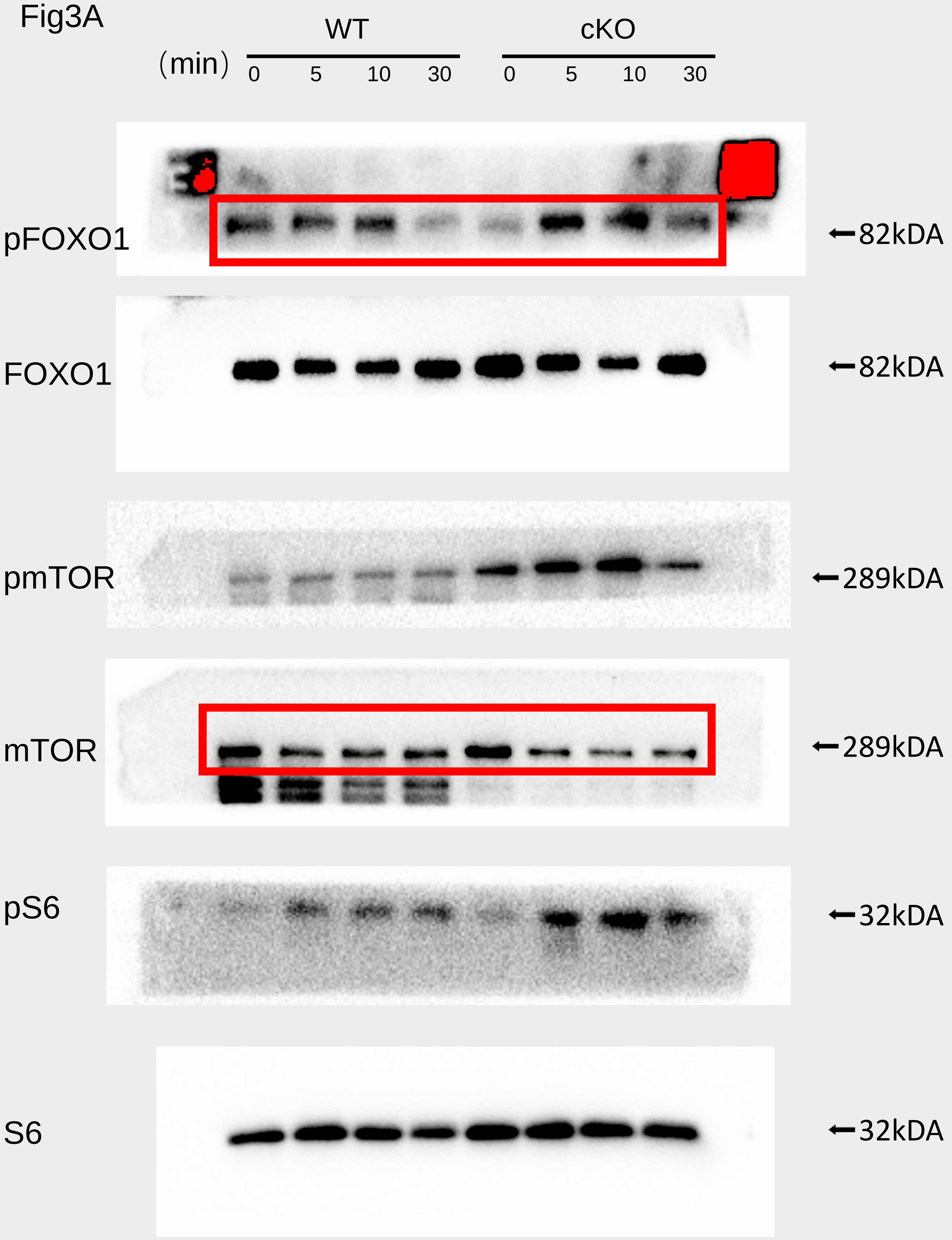

WT
cKO
(min)
0
5
10
30
0
5
10
30
pFOXO1
82kDA
FOXO1
82kDA
pmTOR
289kDA
mTOR
289kDA
pS6
32kDA
S6
32kDA

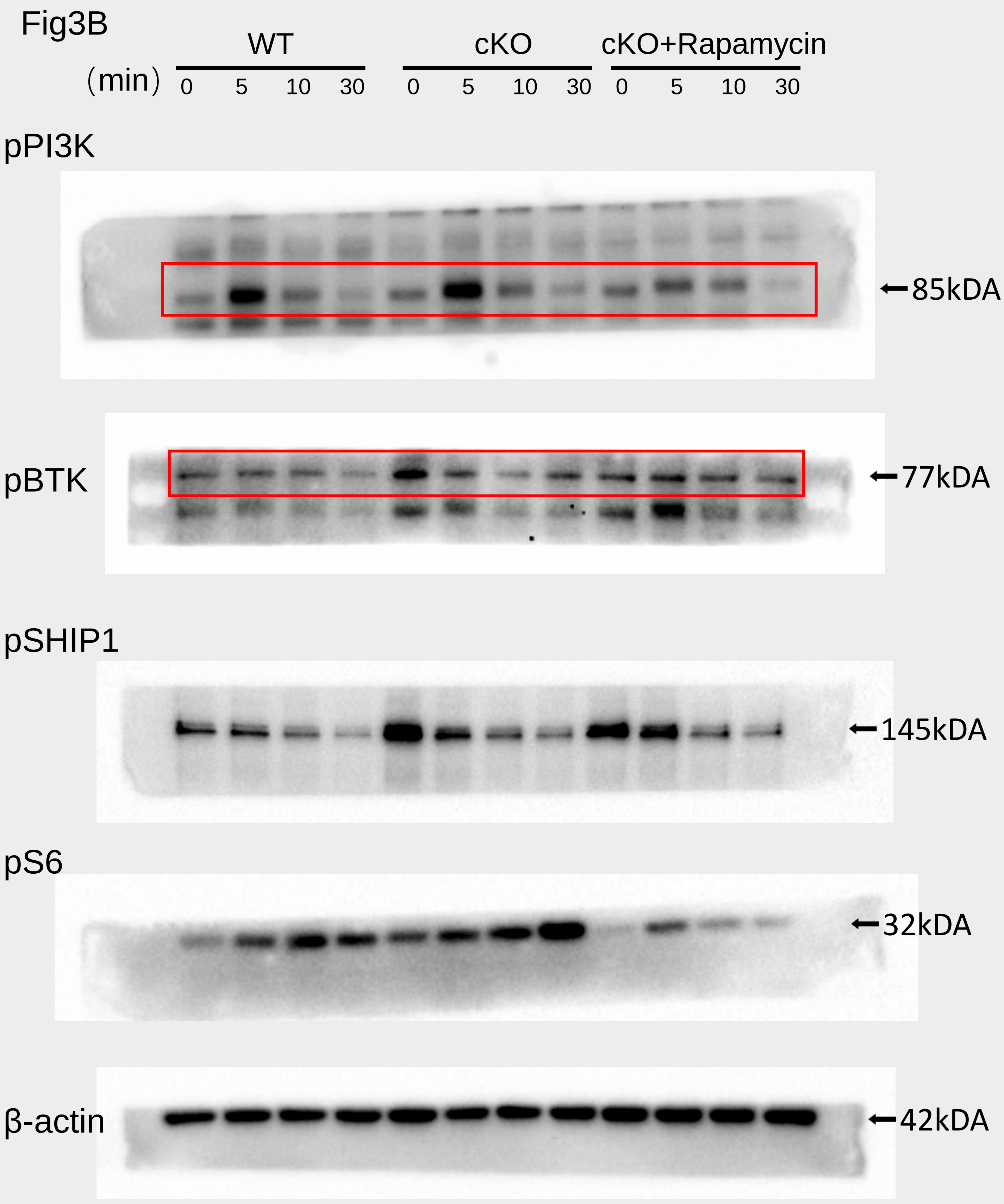
Fig3B
WT
cKO
cKO+Rapamycin
(min)
0 5 10 30 0 5 10 30 0 5 10 30
pPI3K
85kDA
pBTK
77kDA
pSHIP1
145kDA
pS6
32kDA
β-actin
42kDA

Fig3H

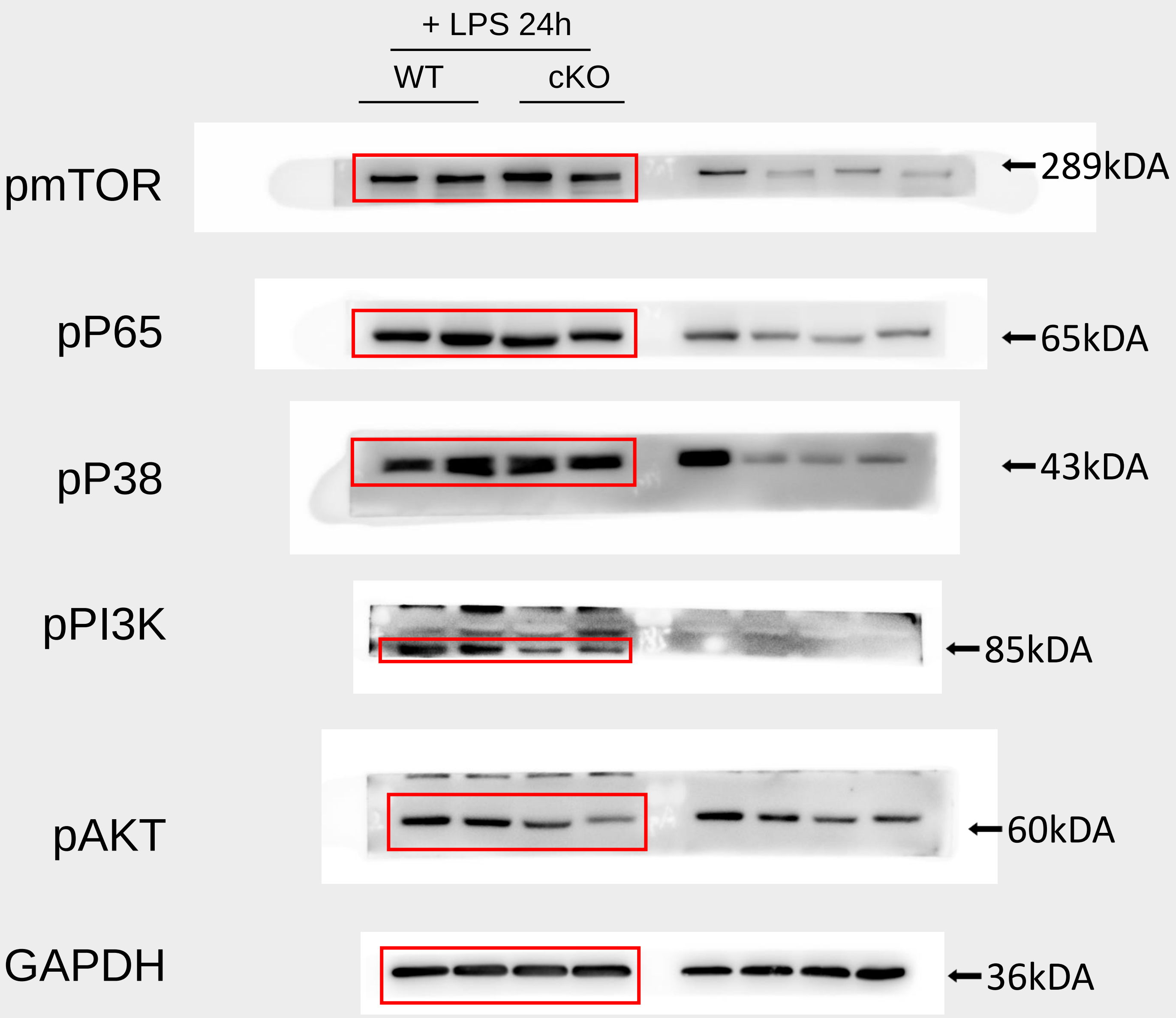
+ LPS 24h
WT
cKO
pmTOR
pP65
pP38
pPI3K
pAKT
GAPDH
289kDA
65kDA
43kDA
85kDA
60kDA
36kDA

Fig3K-1

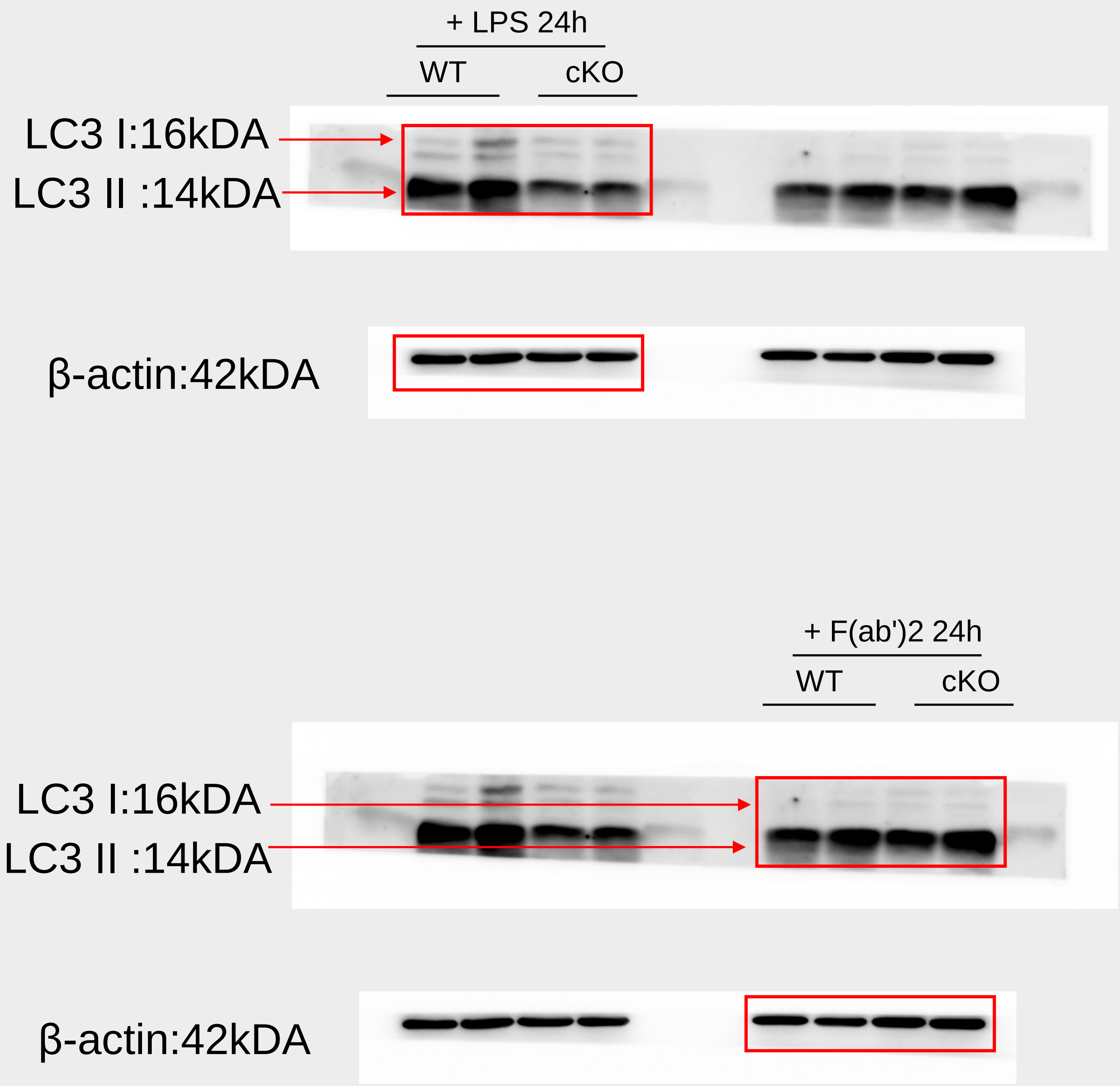
+ LPS 24h
WT
cKO
LC3 I:16kDA
LC3 II :14kDA
β-actin:42kDA
+ F(ab')2 24h
WT
cKO
LC3 I:16kDA
LC3 II :14kDA
β-actin:42kDA

Fig3K-2

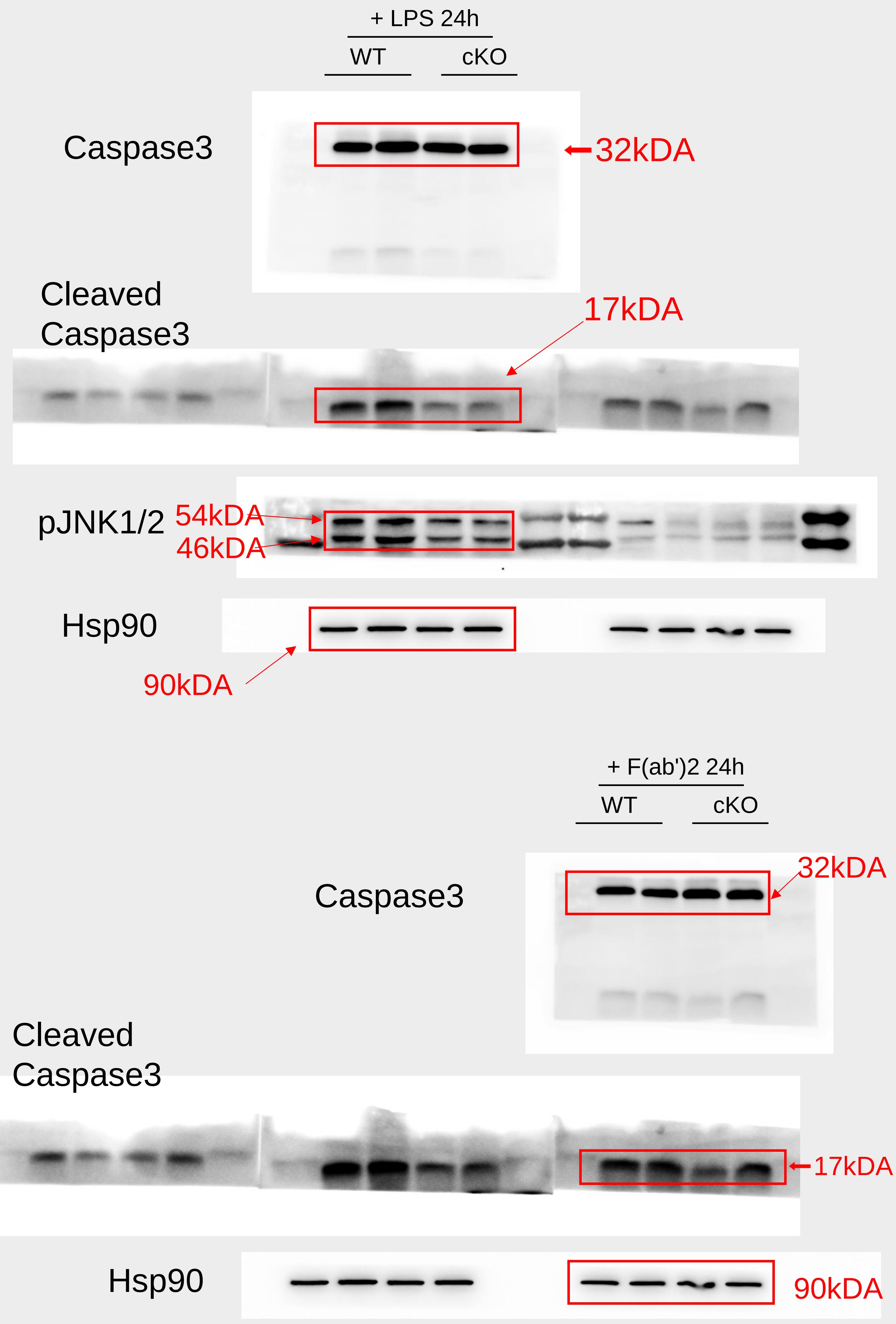

+ LPS 24h
WT
cKO
Caspase3
32kDA
Cleaved Caspase3
17kDA
pJNK1/2
54kDA
46kDA
Hsp90
90kDA
+ F(ab')2 24h
WT
cKO
Caspase3
32kDA
Cleaved Caspase3
17kDA
Hsp90
90kDA

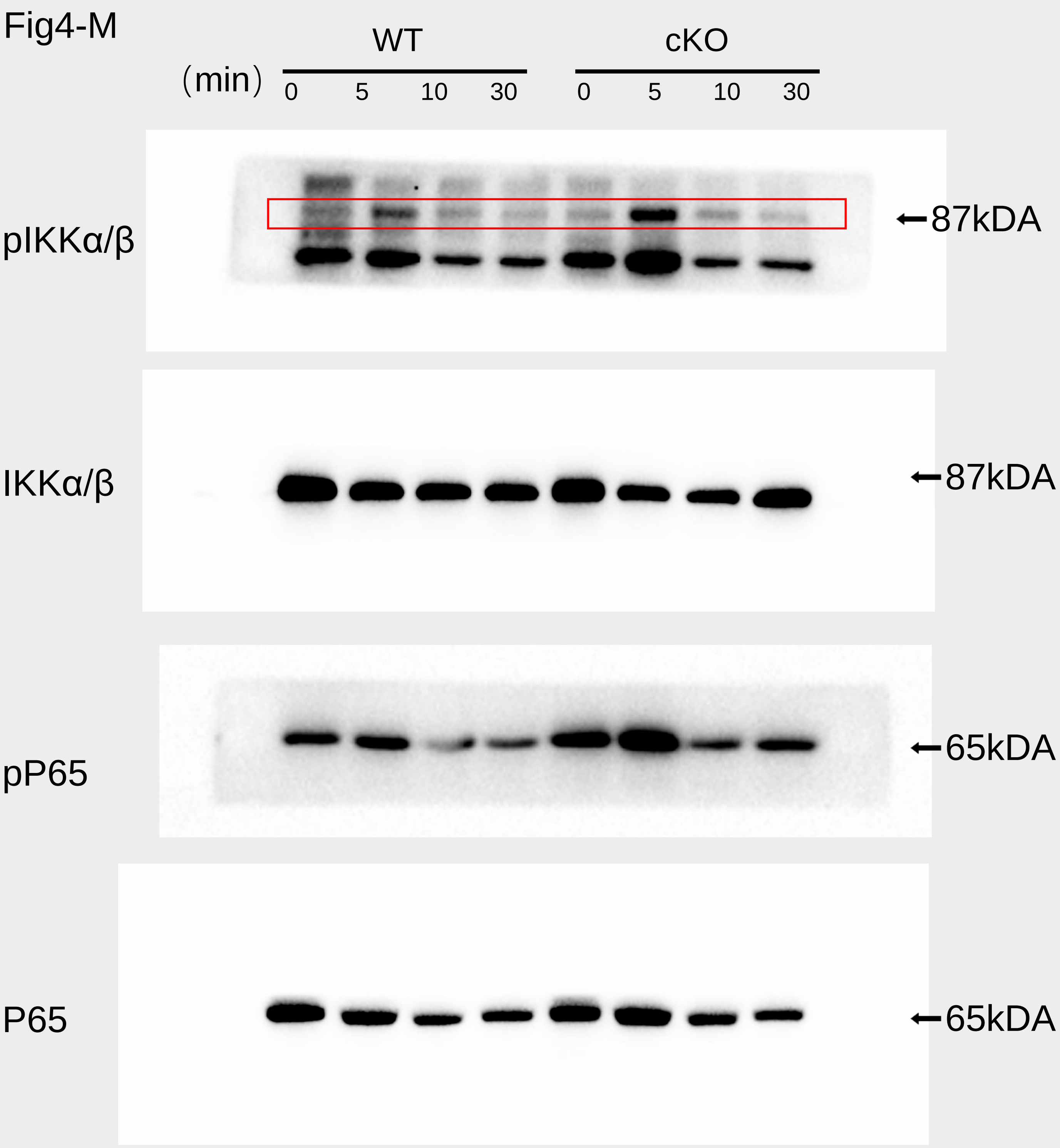
Fig4-M
WT
cKO
（min）
0
5
10
30
0
5
10
30
pIKKα/β
87kDA
IKKα/β
87kDA
pP65
65kDA
P65
65kDA

Fig4-N

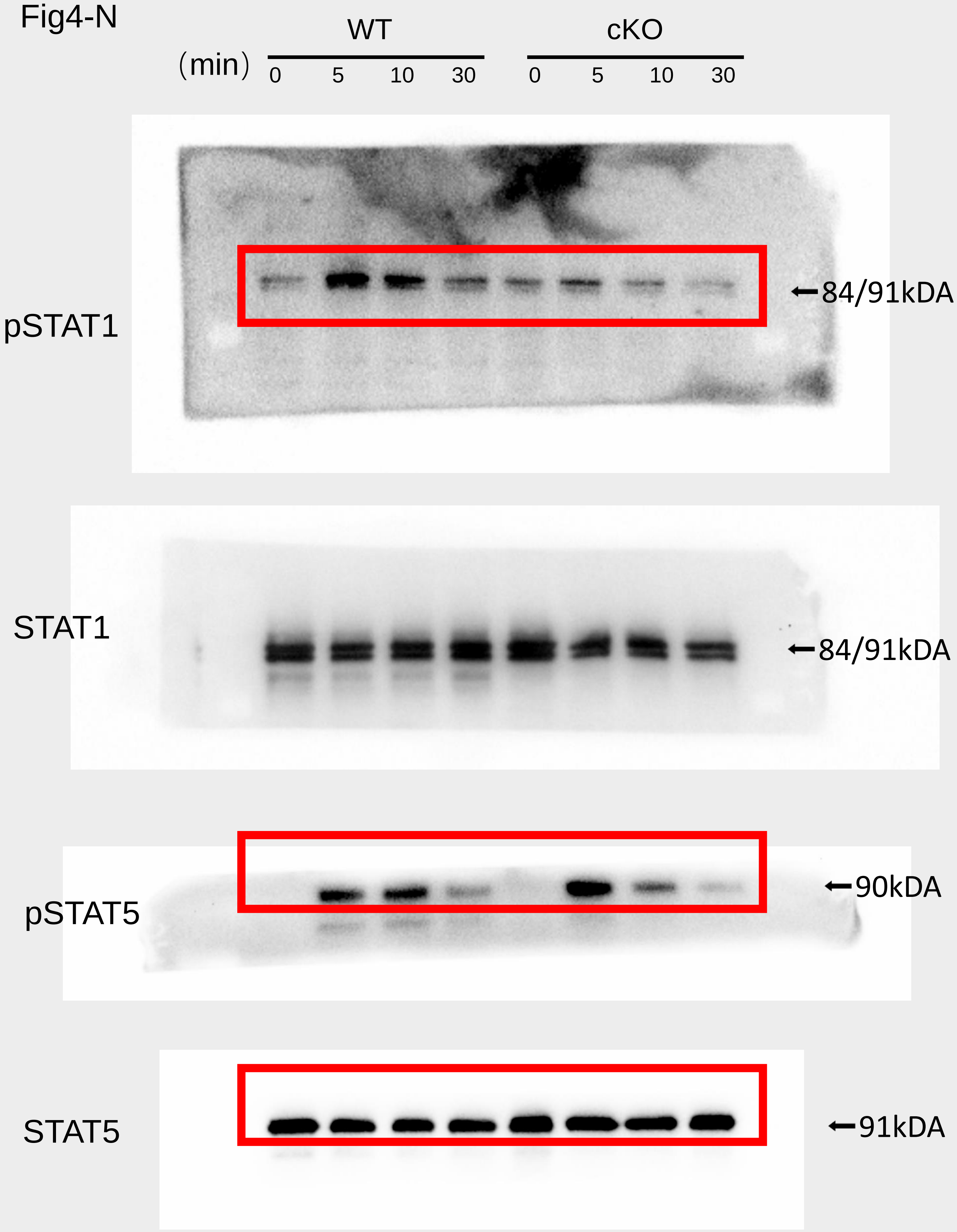
WT
cKO
(min)
0
5
10
30
0
5
10
30
pSTAT1
84/91kDA
STAT1
84/91kDA
pSTAT5
90kDA
STAT5
91kDA

Fig5E

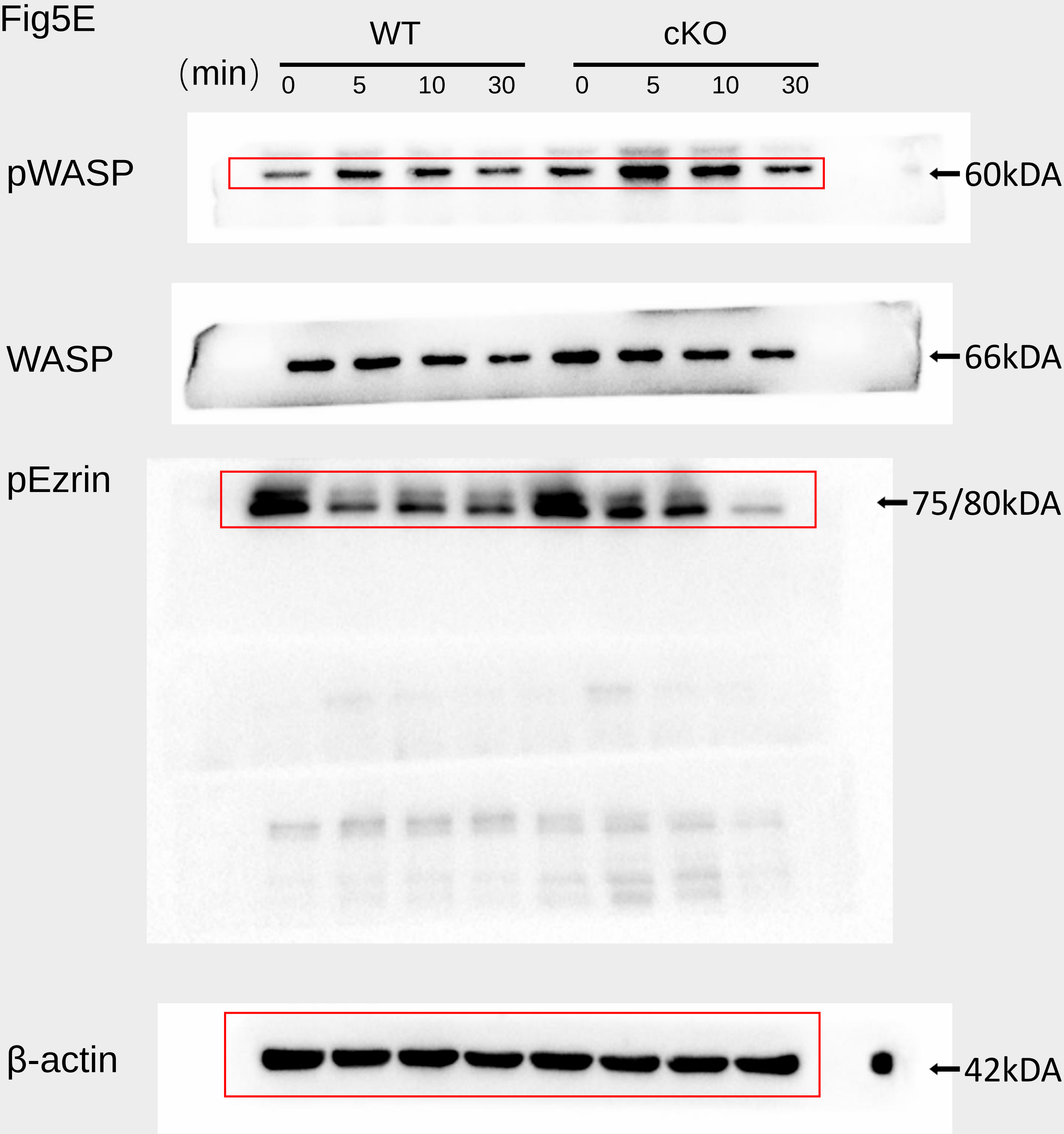

WT
cKO
(min)
0
5
10
30
0
5
10
30
pWASP
60kDA
WASP
66kDA
pEzrin
75/80kDA
β-actin
42kDA

Fig5F

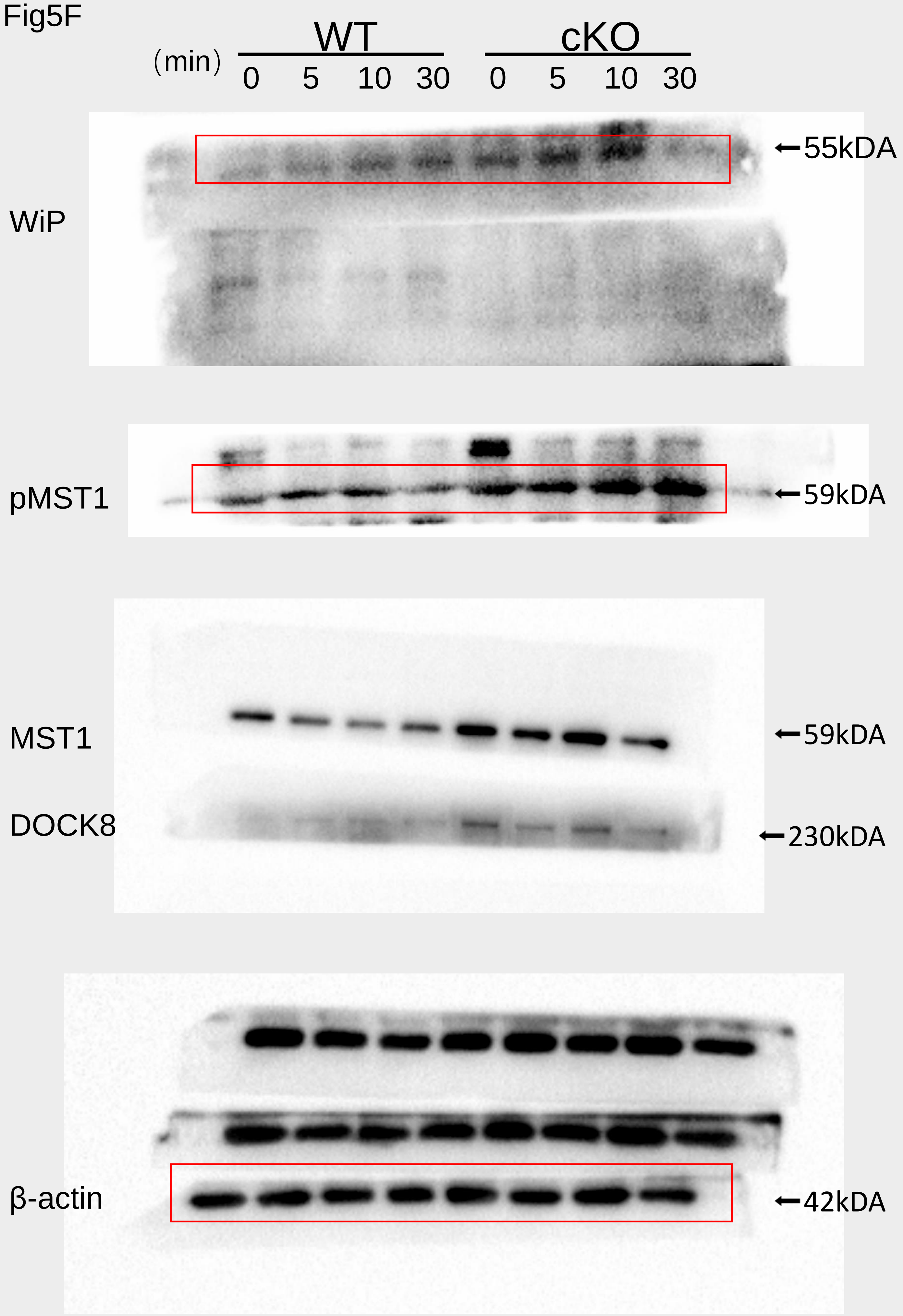
(min)
WT
cKO
0 5 10 30 0 5 10 30
WiP
55kDA
pMST1
59kDA
MST1
59kDA
DOCK8
230kDA
β-actin
42kDA

Fig5G

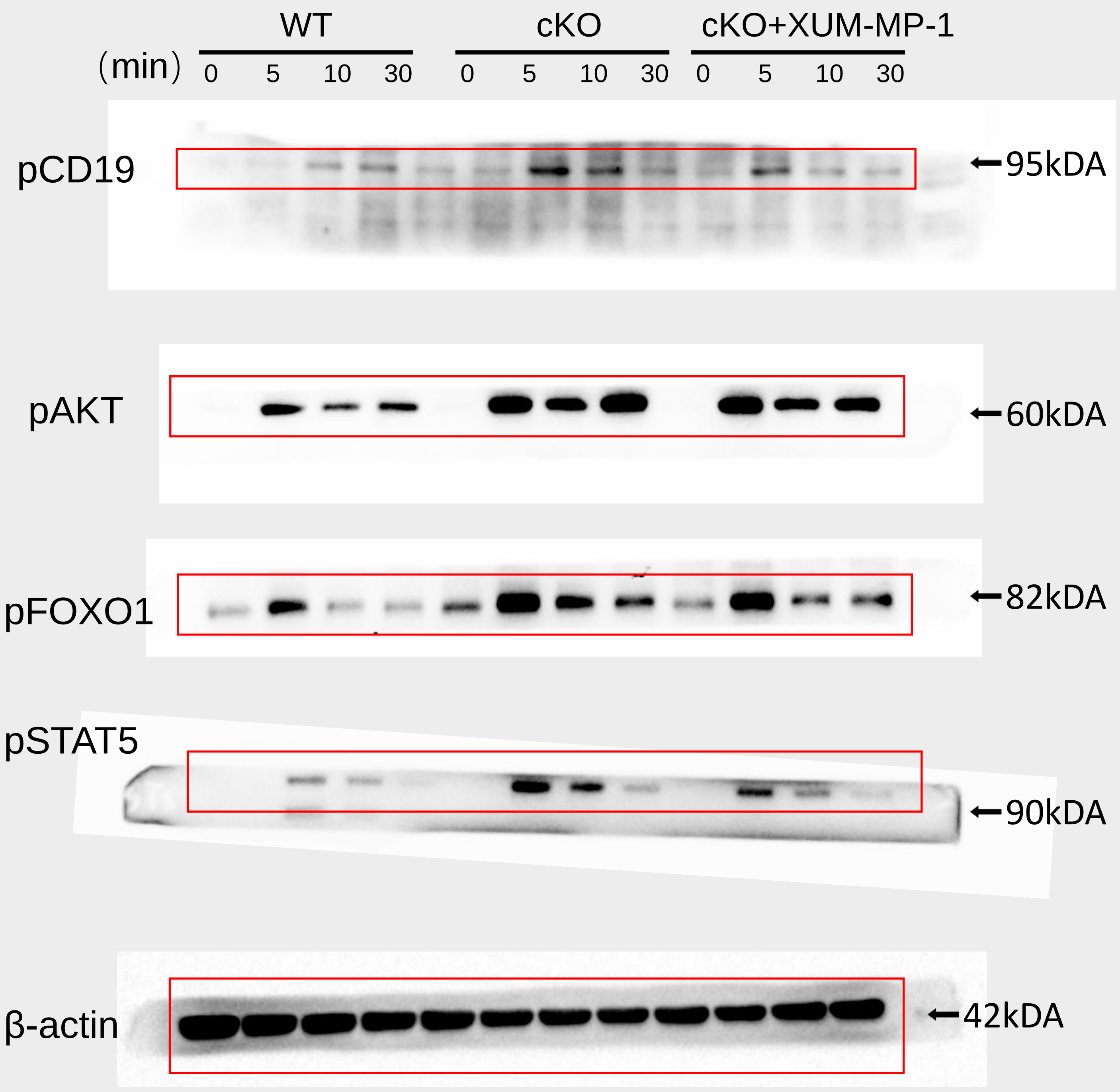

WT
cKO
cKO+XUM-MP-1
(min)
0 5 10 30 0 5 10 30 0 5 10 30
pCD19
95kDA
pAKT
60kDA
pFOXO1
82kDA
pSTAT5
90kDA
β-actin
42kDA

Fig8-K

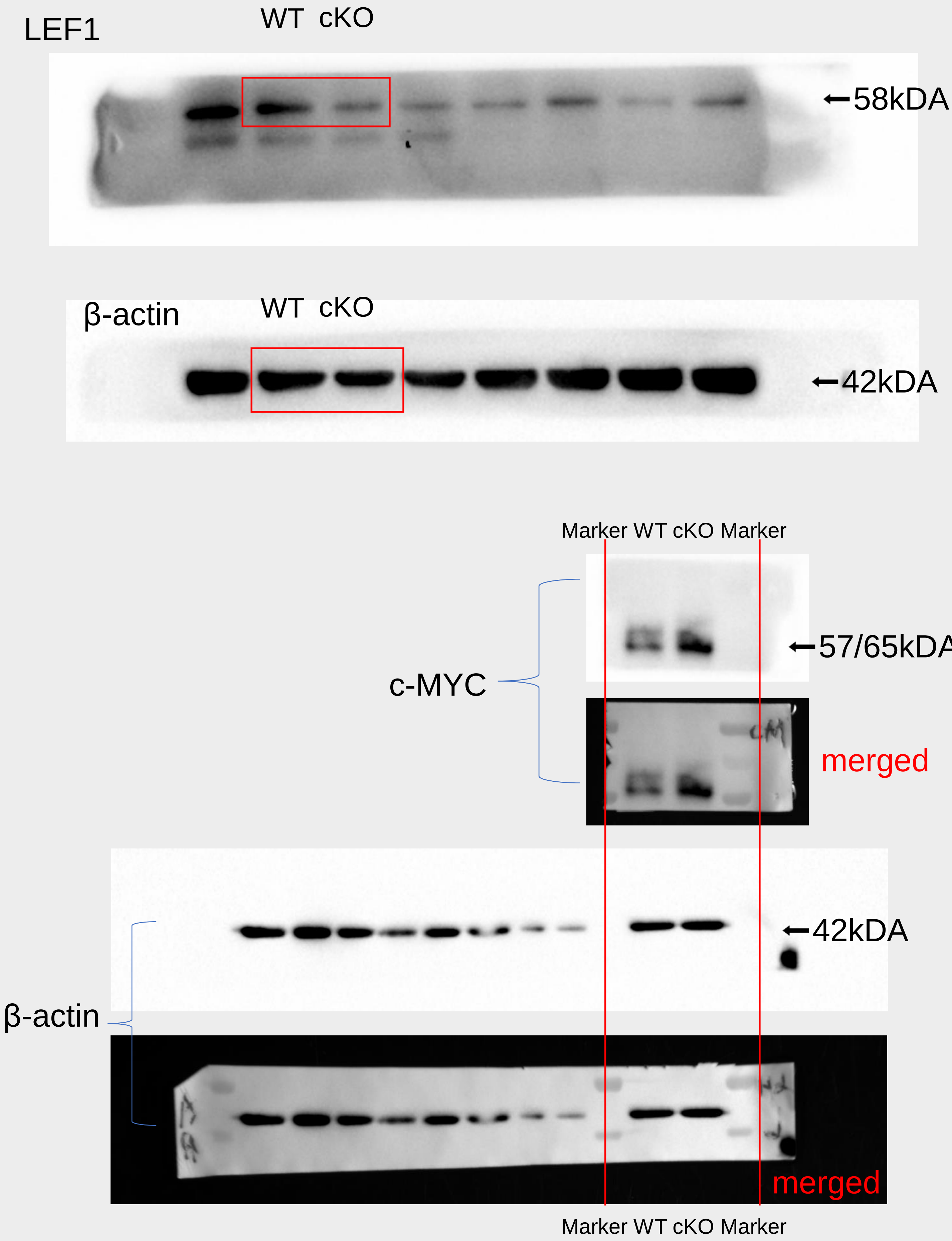
LEF1
WT cKO
58kDA
β-actin
WT cKO
42kDA
Marker WT cKO Marker
c-MYC
57/65kD
merged
β-actin
42kDA
merged
Marker WT cKO Marker

Fig8-M

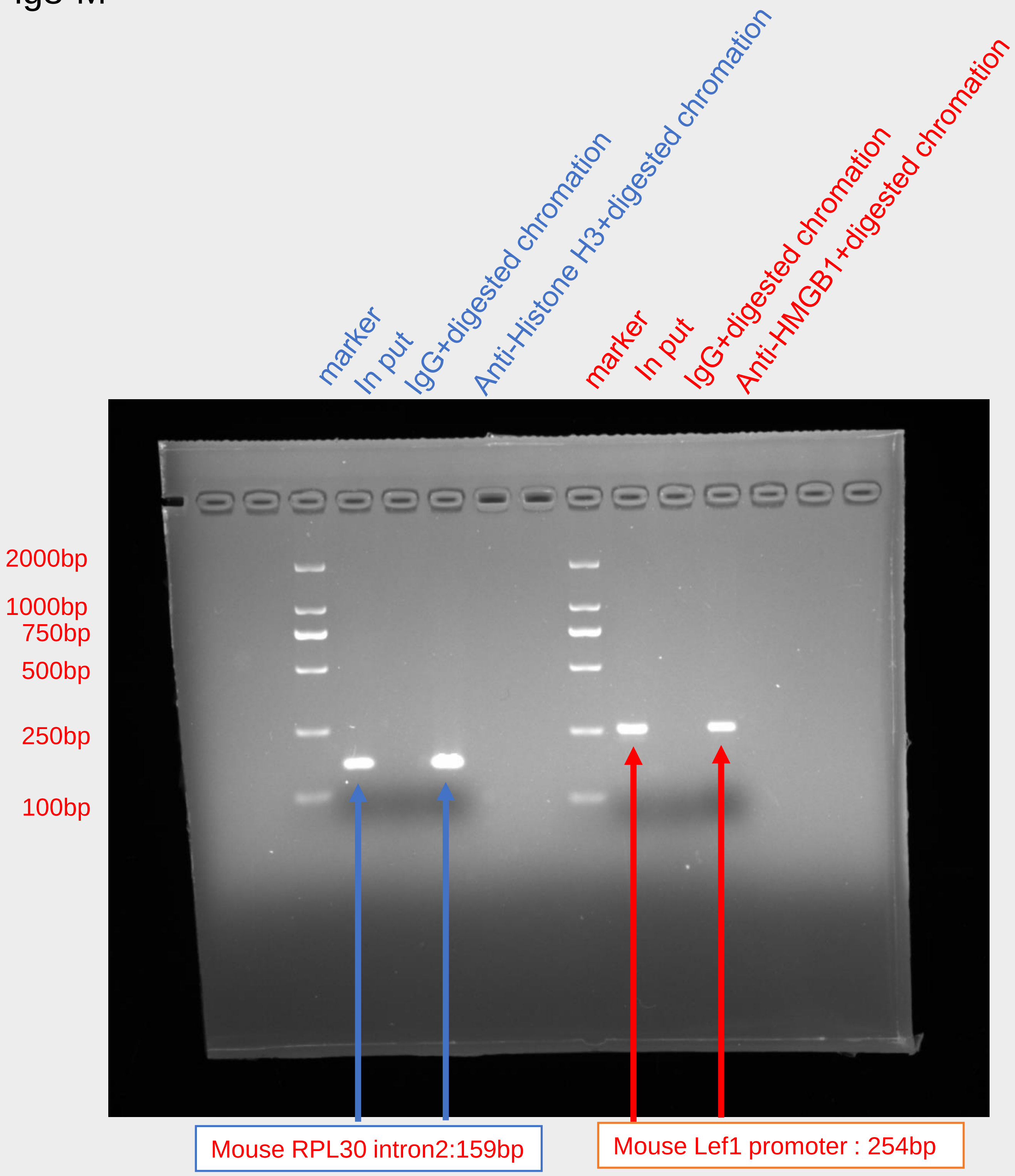
marker
In put
IgG+digested chromation
Anti-Histone H3+digested chromation
marker
In put
IgG+digested chromation
Anti-HMGB1+digested chromation
2000bp
1000bp
750bp
500bp
250bp
100bp
Mouse RPL30 intron2:159bp
Mouse Lef1 promoter : 254bp

Fig S1B

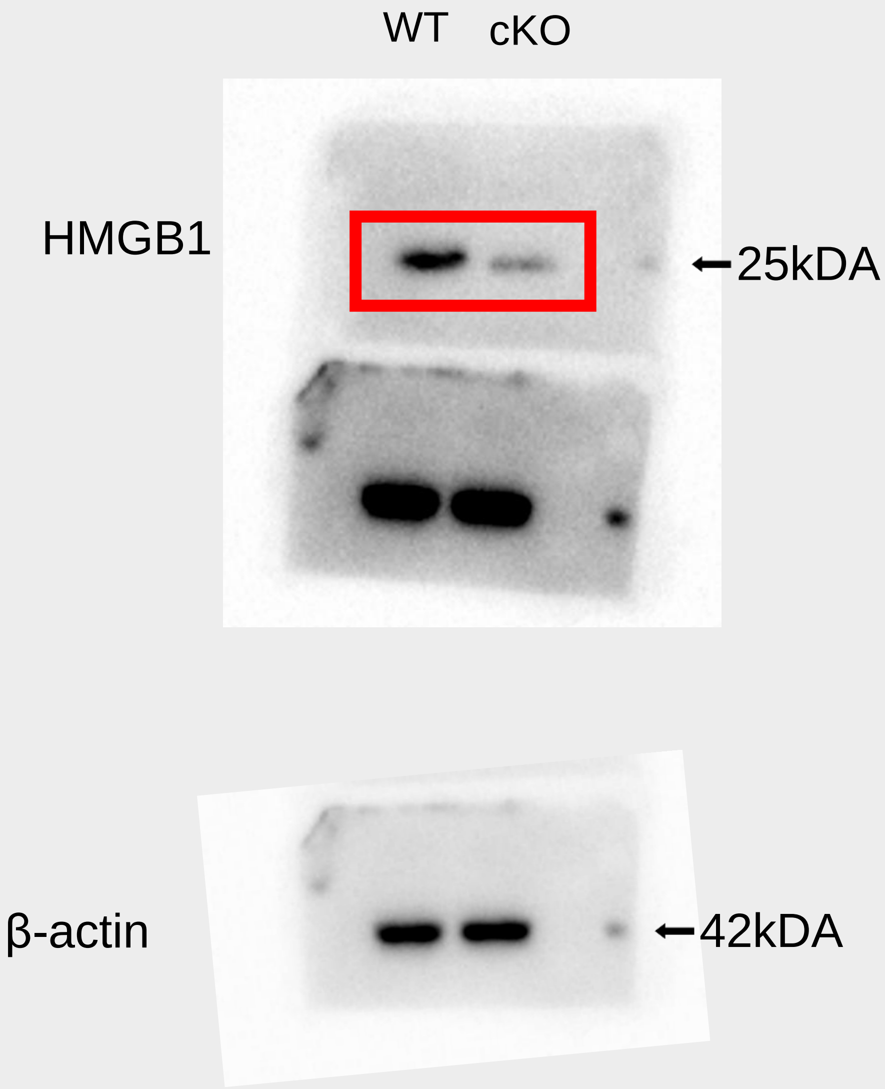

Fig S1-D1

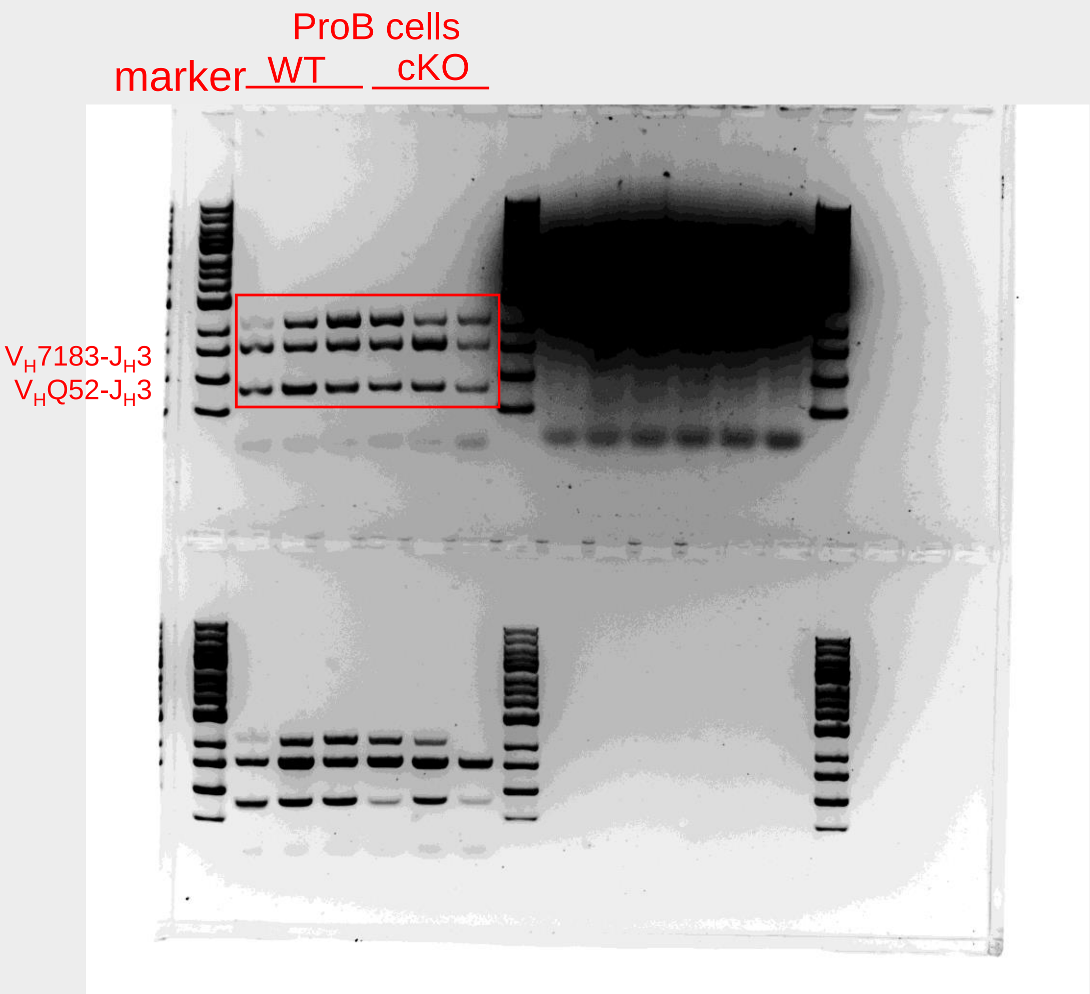
ProB cells
marker
WT
cKO
V_H7183-J_H3
V_HQ52-J_H3

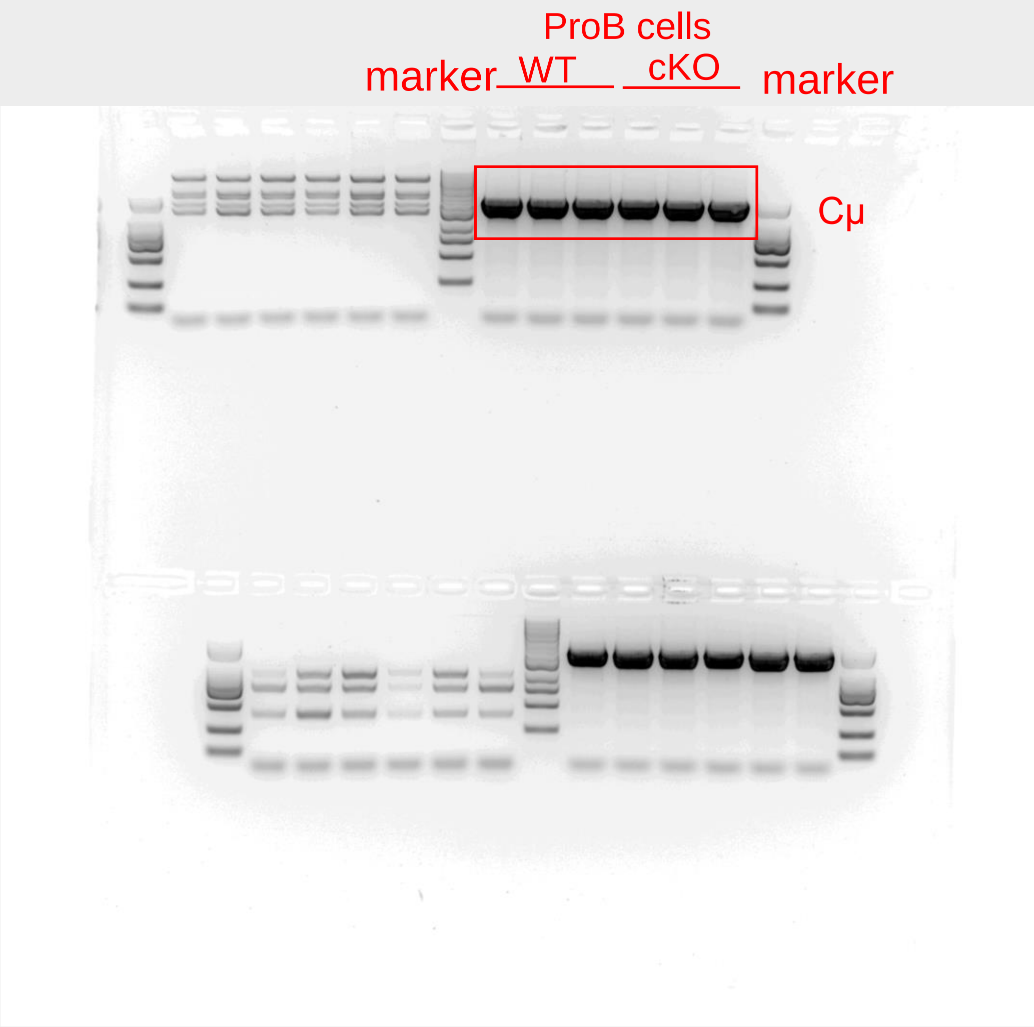
ProB cells
marker
WT
cKO
marker
Cμ

Fig S1-D2

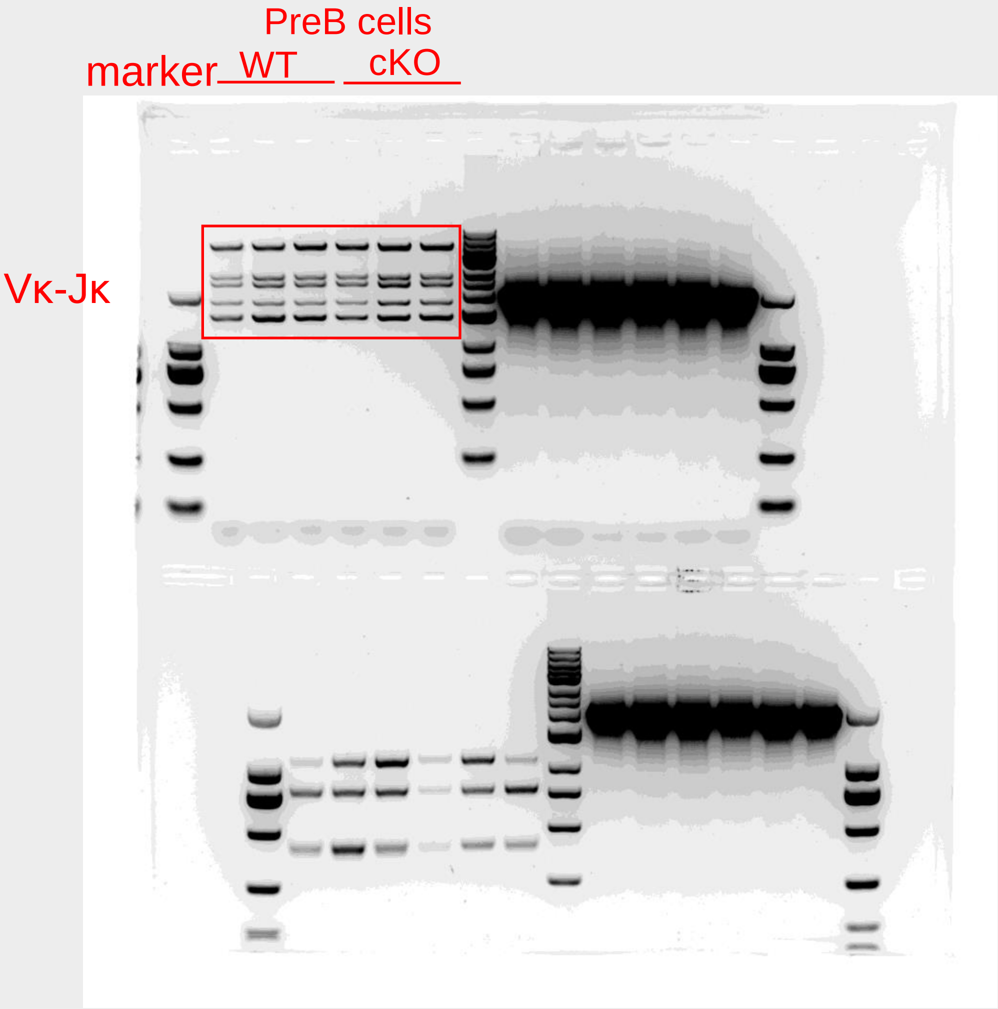
PreB cells
marker
WT
cKO
Vκ-Jκ

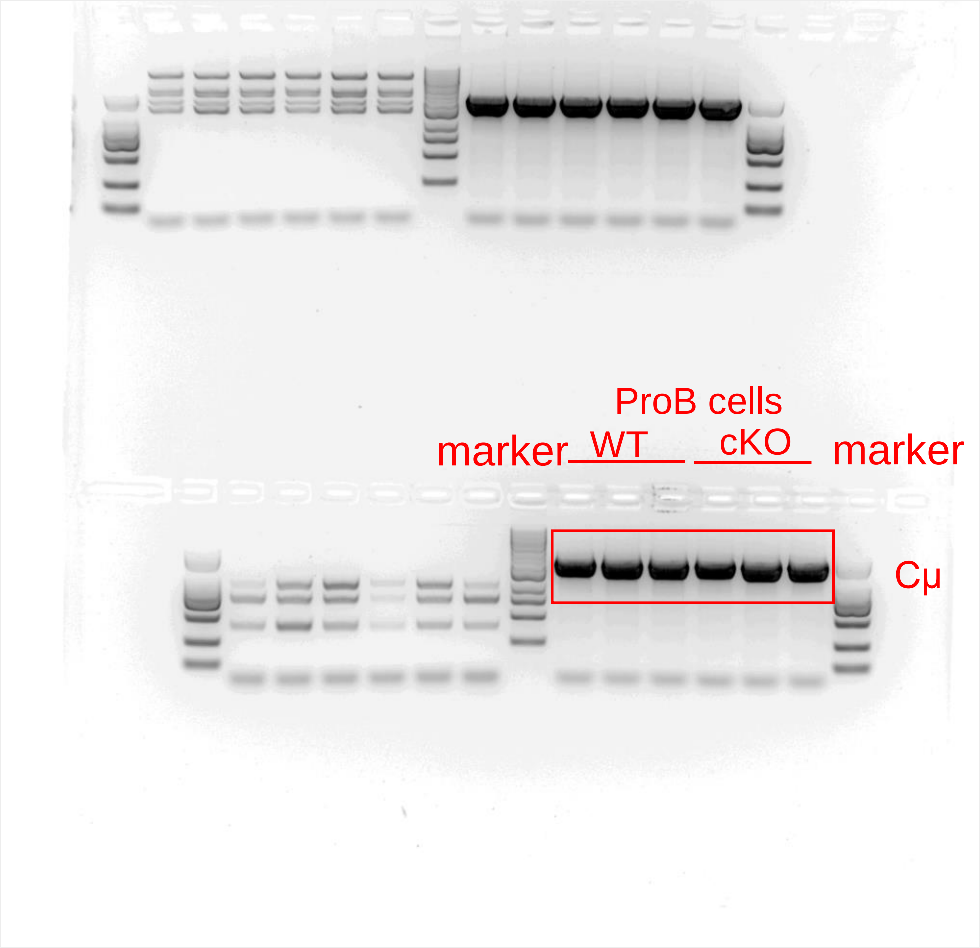
ProB cells
marker
WT
cKO
marker
Cμ

Fig S4-K

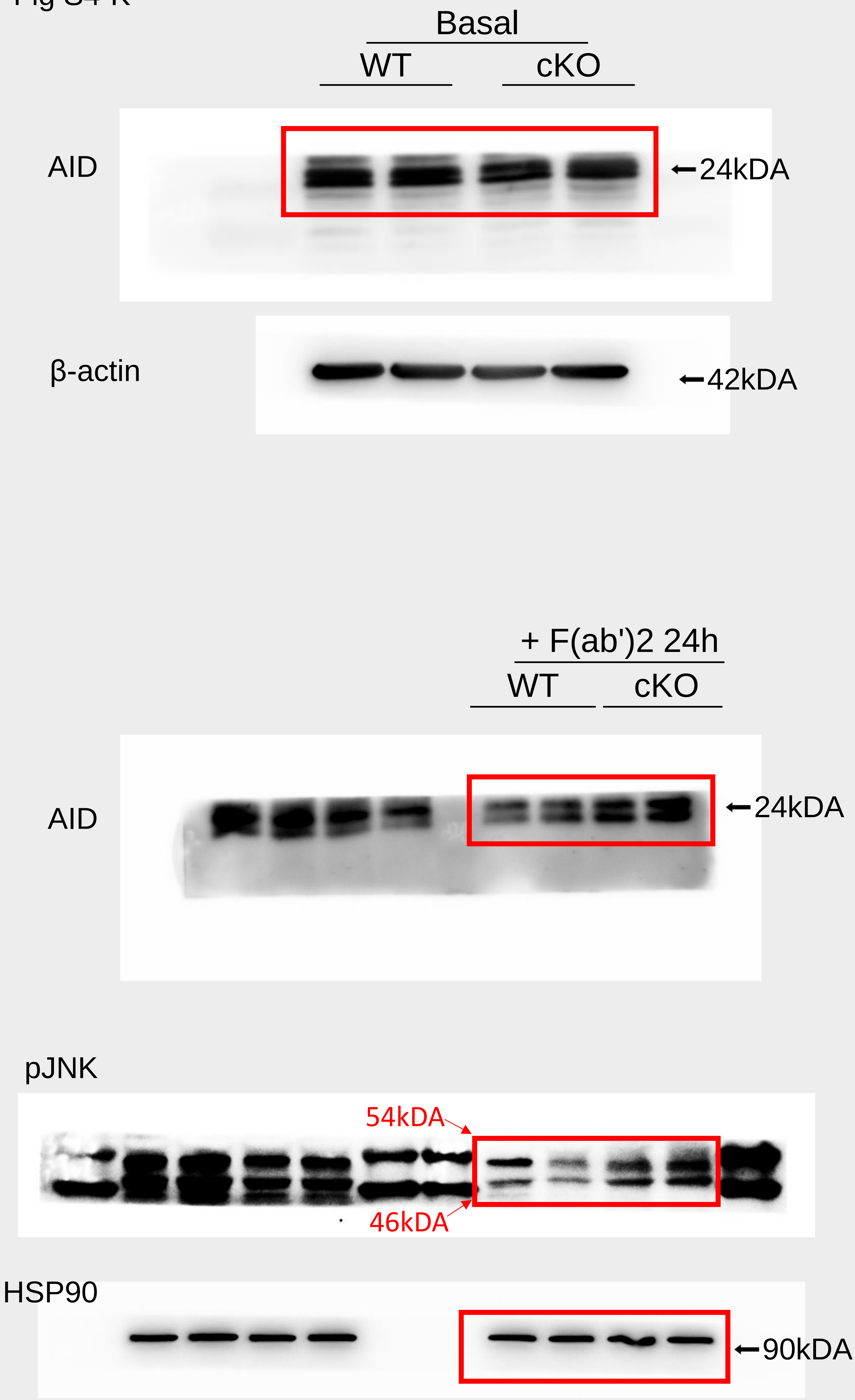

Basal
WT
cKO
AID
←24kDA
β-actin
←42kDA
+ F(ab')2 24h
WT
cKO
AID
←24kDA
pJNK
54kDA
46kDA
HSP90
←90kDA
